# The Open DAC 2023 Dataset and Challenges for Sorbent Discovery in Direct Air Capture

**DOI:** 10.1021/acscentsci.3c01629

**Published:** 2024-05-01

**Authors:** Anuroop Sriram, Sihoon Choi, Xiaohan Yu, Logan M. Brabson, Abhishek Das, Zachary Ulissi, Matt Uyttendaele, Andrew J. Medford, David S. Sholl

**Affiliations:** † Fundamental AI Research, Meta AI, Meta, Menlo Park, California 94025, United States; ‡ School of Chemical and Biomolecular Engineering, Georgia Institute of Technology, Atlanta, Georgia 30332, United States; ¶ Oak Ridge National Laboratory, Oak Ridge, Tennessee 37831-2008, United States

## Abstract

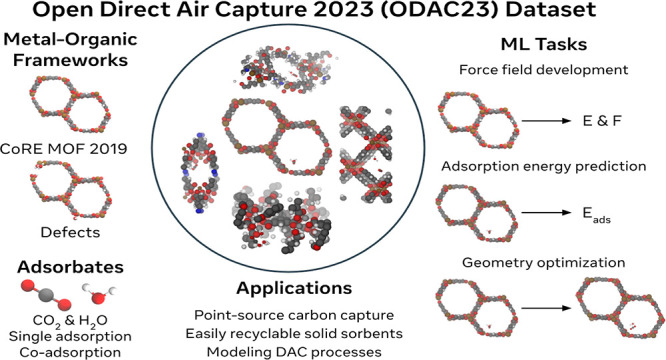

Direct air capture (DAC) of CO_2_ with porous adsorbents such as metal−organic frameworks (MOFs) has the potential to aid large-scale decarbonization. Previous screening of MOFs for DAC relied on empirical force fields and ignored adsorbed H_2_O and MOF deformation. We performed quantum chemistry calculations overcoming these restrictions for thousands of MOFs. The resulting data enable efficient descriptions using machine learning.

## Introduction

Annual anthropogenic carbon emissions reached nearly 36 billion tonnes in 2020, and the atmospheric carbon dioxide concentration has increased ∼50% since preindustrial times to approximately 420 ppm.^1^ Rising CO_2_ levels have motivated the development of carbon capture and sequestration (CCS) technologies to combat the effects of emissions on global climate change.^2^ Direct air capture (DAC) is an emerging technology with the potential for distributed capture and negative emissions.^3^ DAC operates at ambient conditions and avoids impurities that are common for point source capture of CO_2_, but the low concentration of CO_2_ requires the movement of large volumes of air and strong adsorption of CO_2_.^4^ Many current DAC absorbents, such as liquid amines and solid alkali hydroxides, strongly bind CO_2_ through chemisorption, requiring energy-intensive regeneration of the sorbent.^5,6^ Metal–organic frameworks (MOFs) are a promising class of alternative sorbent materials for DAC allowing regeneration at relatively low temperatures. In contrast to sorbents such as alkali hydroxides, MOFs are modular, flexible, and highly tunable, and they possess remarkably high porosities, low densities, and long-range order.^7^ Their chemical tunability and long-range order make MOFs worthy of high-throughput computational screening studies.

Computational materials design is a promising strategy for DAC sorbents.^8^ Design of efficient DAC processes may require tailoring of materials to the specifics of the air temperature and humidity conditions in a given environment or the temperature/pressure swings that are required to keep energy consumption low.^9^ This is particularly true of DAC processes that seek to leverage air movement and energy content of existing systems such as heating, ventilation, and air conditioning.^10^ The consideration of humidity is particularly important since dehumidifying air requires significant energy input, the presence of H_2_O can result in competitive adsorption even at low relative humidities, and humidity can in some cases cause adsorbent degradation over time.^11−14^ The availability of large datasets of MOFs and other solid sorbent materials can facilitate the identification of specific materials or chemical moieties that are well suited for the specific conditions of a given DAC process.^15,16^

High-throughput computational studies and machine learning (ML) techniques are already a common practice in the screening and discovery of MOFs and other reticular materials.^12,17−25^ There are several large databases of MOF^26−31^ and zeolite structures^32^ and multiple computational toolkits^33−37^ and ML models^23,38−43^ to analyze and predict the adsorption properties of these materials. However, there are several key limitations to the existing body of work. First, because of the computational costs involved, many studies rely on empirical force field (FF) models for predicting adsorption properties. Inaccuracies associated with FFs can lead to both qualitative and quantitative inconsistencies in the prediction of material performance, particularly in the case of open-metal sites (OMS) or defects where covalent bonding or complexation occurs.^17,44−49^ There are several large databases of density functional theory (DFT) calculations for MOF materials,^27,31^ but to date these are focused only on the MOF structure and do not include adsorption data. Second, many existing databases and studies of CO_2_ adsorption focus only on adsorption of CO_2_, neglecting the possibility of competition with H_2_O.^11,50−53^ Failure to consider competitive adsorption will strongly limit the ability to predict materials for practical DAC processes, where bicomponent CO_2_/H_2_O isotherms are required. Accurately modeling H_2_O adsorption with classical FFs is challenging due to the complex physical properties of water.^54−57^ Third, many computational databases and studies focus on hypothetical materials,^28,30,58,59^ which leads to practical challenges in the synthesis and experimental testing of new predicted materials. Finally, most datasets are restricted to pristine materials. In reality, MOFs will contain a wide range of defects that may govern their adsorption properties under practical conditions.^60,61^ New materials can also be created by inserting defects in MOFs via so-called defect-engineering.^62^ Large datasets of high-quality DFT simulations of mixed CO_2_ and H_2_O adsorption on realistic pristine and defective MOFs are needed to address these limitations.

ML is also a well-established approach in the discovery of MOFs and other nanoporous materials. ML models have been applied to directly predict the adsorption properties and isotherms of MOFs based on their physical and chemical structures.^23,41−43,63−65^ Descriptors based on the porosity, chemical constituents, and energy landscape of probe adsorbates in MOFs have been combined with a range of regression and classification models to provide predictions of gas loadings,^23,42,66,67^ Henry’s constants,^40,68^ and temperature-dependent isotherms.^63,64^ Neural networks have been used to predict MOF properties and perform inverse design tasks to identify MOF materials with high thermal stability^58,69^ and strong or selective CO_2_ adsorption.^43,59,65^ ML models have also been trained to provide insight into the synthesizability and stability of MOFs and zeolites.^70−74^ However, the training data required for many of these properties, such as adsorption isotherms, are generated using classical FFs, which have been shown to exhibit systematic errors.^45^ Efforts to train ML models that can directly emulate DFT data for MOFs are more limited.^43^ The ability to use ML models to directly replace FFs in MOFs has the potential to enhance many of the prior efforts.

**Figure 1:**
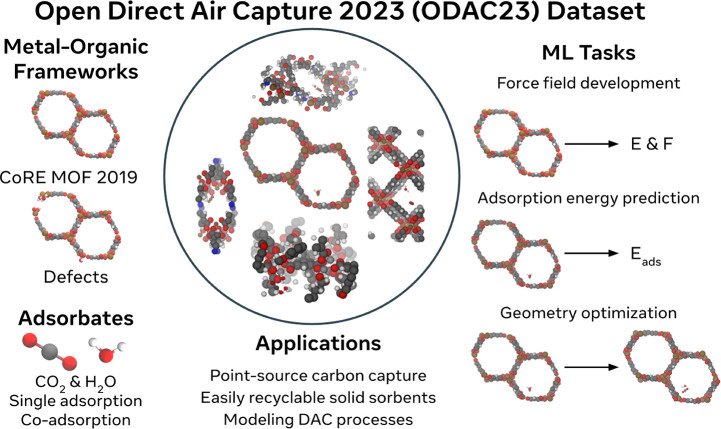
Materials, adsorbates, tasks, and potential applications of the ODAC23 dataset. Images are randomly sampled from the dataset.

In this work, we introduce the Open DAC 2023 (ODAC23) dataset to address these challenges. The dataset consists of adsorption energies for CO_2_, H_2_O, and mixtures thereof on ∼8K MOFs, amounting to a total of ∼176K adsorption energies and ∼38M single-point calculations (Figure 1). All calculations were performed using DFT with the PBE+D3 exchange correlation functional, ensuring that covalent and electrostatic interactions are treated quantum mechanically and van der Waals interactions are included with well-established empirical accuracy. Approximately 76K adsorption energies involve MOFs that have missing linker defects, providing a route to predict the role of defects. The dataset is used to train and evaluate state-of-the-art ML models for the prediction of adsorption energies and atomic forces using approaches developed for the Open Catalyst Project.^75^ In addition, we include several out-of-domain datasets taken from the extended CoRE MOF database^58,76^ to evaluate the ability of the trained models to generalize to unseen topologies and linker chemistries. We expect that this dataset and the associated infrastructure will accelerate the development of MOF materials for DAC by providing a common dataset that far exceeds the size of any currently available dataset, establishing well-defined standards and benchmarks for the development of new ML models, and providing accessible pretrained ML models that enable routine prediction of mixed CO_2_ and H_2_O adsorption on MOFs at an accuracy that approaches DFT.

The ODAC23 dataset is publicly available at the OpenDAC Web site.^a^ All of our trained ML models and training code are available in the OCP repository.^b^

### Scope and Structure of the ODAC23 Dataset

Enormous numbers of hypothetical MOF structures exist, as illustrated by the hypothetical MOF database (hMOF) of Wilmer et al., which contains 138,000 structures.^28^ Several other MOF databases have been developed, including the Topologically Based Crystal Constructor (ToBaCCo) database of 13,512 MOFs with 41 unique topologies developed by Colón et al.^30^ Perhaps most importantly, Chung et al. developed the Computation-Ready, Experimental (CoRE) MOF database^26^ and its 2019 expansion^27^ from experimentally synthesized structures in the Cambridge Structural Database (CSD).^77^ The CoRE MOF database has been the foundation of many studies and extensions, including assignment of DFT-derived point charges,^78^ more thorough cleaning by removal of structures with misbonded or overlapping atoms,^79^ and the QMOF database of DFT-derived properties of many CoRE MOF structures.^31^

The Open DAC dataset uses the CoRE MOF 2019 work as a starting point. This approach is beneficial because the data are readily available, and the origin of each MOF in the database in an experimentally reported synthesis partially addresses concerns surrounding practicality when considering candidate MOFs for experimental testing. The CoRE MOF database has also been shown to be more chemically diverse than larger databases of hypothetical materials, which is beneficial for training transferable and generalizable ML models.^80^ The CoRE MOF 2019-ASR database contains 12,020 unique structures with accessible data. We considered only MOFs that contain fewer than 1,000 atoms in the unit cell due to computational cost. MOFs with a pore limiting diameter (PLD) of less than 3.3 Å are excluded because a CO_2_ molecule (kinetic diameter of 3.3 Å) may experience kinetic limitations in entering such small pores.^27^ With these limitations, 8,803 MOFs serve as our starting point for DFT relaxation.

We used the Perdew–Burke–Ernzerhof functional^81^ with a D3 dispersion correction^82,83^ (PBE-D3) for all calculations. The generalized gradient approximation (GGA) approach was chosen over more accurate methods such as hybrid functionals or coupled cluster techniques because of the size and diversity of the dataset. Nazarian et al. showed that several different functionals and dispersion corrections perform similarly when making structural and partial charge predictions on a chemically diverse set of MOFs.^78^ We did not include a Hubbard *U* correction. Without this correction, PBE systematically overpredicts binding energies on open-metal sites, but *U* values are empirical and are difficult to find for every metal type.^84^ Our calculations included spin polarization to capture spin effects associated with open metal sites, with the simplification that the magnetic moment was initialized as +1 for all atoms. Further exploration of possible spin states may be warranted in cases of special interest. Our work ultimately seeks to push the baseline description of MOFs for DAC from classical FFs to the PBE-D3 level of theory, so we prioritized consistency across a very large number of calculations rather than absolute accuracy.

The ODAC23 dataset consists of complete relaxation trajectories of CO_2_, H_2_O, and mixtures of CO_2_ and H_2_O in MOF structures derived from the CoRE MOF database. We include two classes of MOF frameworks: *pristine* frameworks and *defective* structures. Although a range of defect types can exist in MOFs, we only considered missing linker defects, since systematic methods exist to add these defects to MOF structures.^85^ Pristine MOF structures are obtained from the CoRE MOF database without further modification. Approximately 66% of the pristine MOFs include frameworks with open metal sites. To test generalizability, we also included 114 “ultrastable” MOFs from Nandy et al. created by fragmenting and recombining linkers and nodes from the original CoRE MOF database.^76^ The final data set includes a total of 4,942 pristine MOFs and 3,470 defective MOFs with defect concentrations ranging from 1 to 16%. The MOFs contain a diverse set of 57 metals, with Zn, Cu, and Cd being the most common, and include a mix of monometallic (89%), bimetallic (10.7%), and trimetallic (<1%) frameworks. The abundance of various metals is provided in Table S1, and the most common linkers are listed in Table S2. The adsorbates were initially placed using classical FFs and Monte Carlo sampling, with ∼2–6 placements per framework. The selection of MOFs and adsorption configurations included in the final set are established by pragmatic constraints and practical considerations. In total, the dataset consists of over 170K converged adsorption energies and nearly 40M single point calculations, corresponding to over 400M core-hours of compute time. Details are provided in the Methods section.

The ODAC23 dataset has been designed to allow training of ML models to approximate DFT calculations, similar to previous work in heterogeneous catalysis (OC20 and OC22).^75,86^ We use the same three task definitions used in the OC20 work. These tasks are briefly summarized below, and we refer the reader to the OC20 paper^75^ for more detailed descriptions.

In each task, the input structure is a unit cell periodic in all directions containing a MOF with one or more adsorbates. The ground truth targets of forces, energies, and relaxed structures were all calculated using DFT. For energy targets, we used a nonrelaxed adsorption energy:
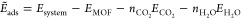
1where *E*_system_ is the energy of the MOF and adsorbates, *E*_MOF_ is the energy of the relaxed MOF structure without an adsorbate, *n*_*i*_ is the number of adsorbate *i*, and *E*_*i*_ is the energy of adsorbate *i* in the gas phase. The tilde on  denotes that *E*_system_ is not necessarily a relaxed structure. In specific cases where *E*_system_ is relaxed, the tilde is dropped, and the adsorption energy is denoted as *E*_ads_. More details are provided in the Methods section.

The energies of these MOF + adsorbate structures were used to train models for three tasks:

1. **Structure to Total Energy and Forces (S2EF)** takes a structure as input and predicts  of the system as well the force on each atom. This task is analogous to training a force field for all atoms in the system.
2. **Initial Structure to Relaxed Energy (IS2RE)** takes an initial guess structure as input and predicts *E*_ads_ of its relaxed structure. This task is analogous to predicting an adsorption energy from an initial structure.
3. **Initial Structure to Relaxed Structure (IS2RS)** takes an initial guess structure as input and predicts the relaxed position of each atom. This task is analogous to geometry optimization.

**Figure 2:**
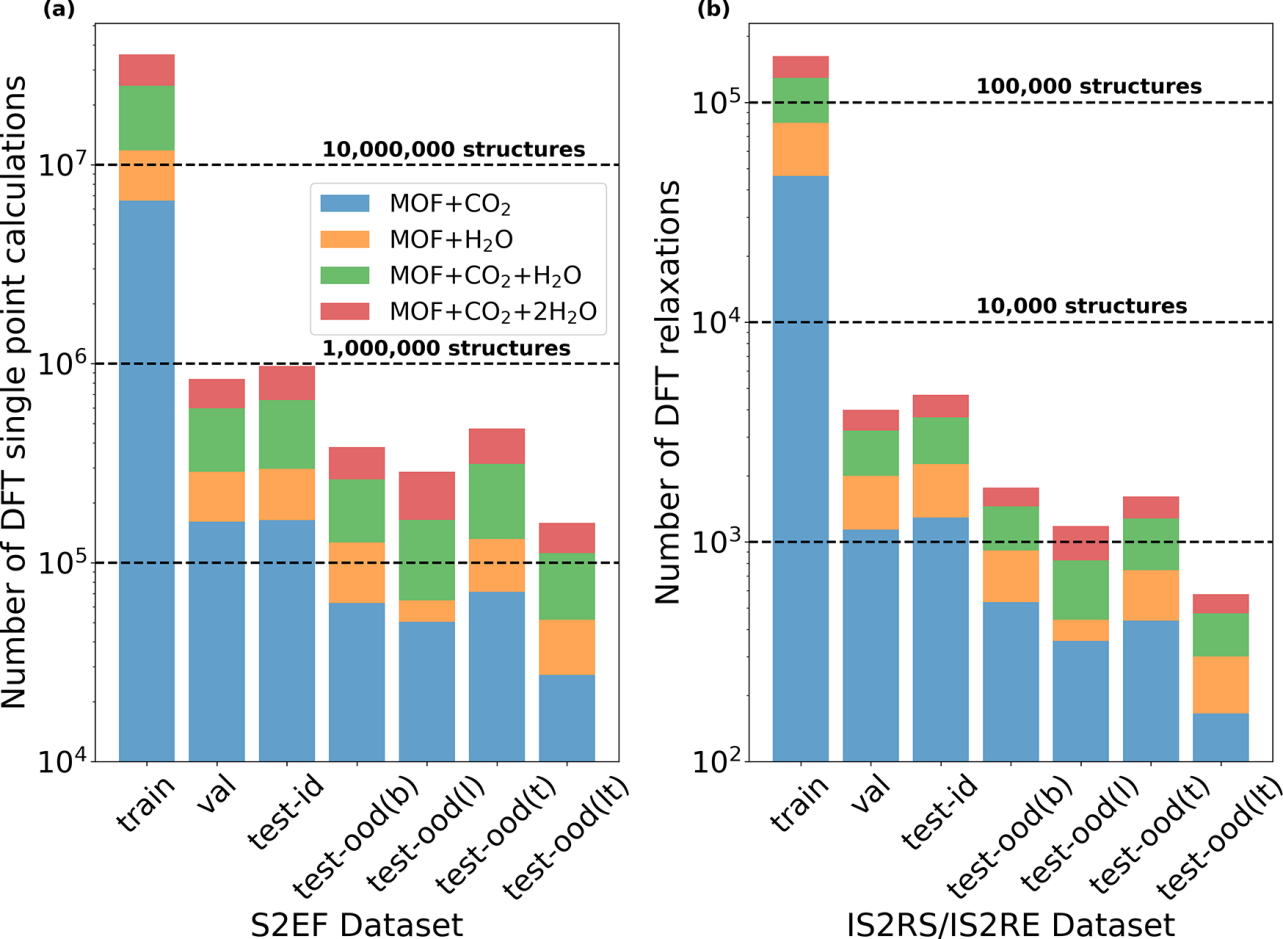
Distribution of the number of MOF + adsorbate DFT calculations for the (a) S2EF and (b) IS2RS/IS2RE tasks on a logarithmic scale. The horizontal lines emphasize the size of the dataset.

The S2EF task is the most general, and an S2EF model can be used to complete the IS2RS and IS2RE tasks. The data set is organized by task and train/test splits. For each task, the data are split into a training set, testing set, and validation set. These in-domain (id) sets are randomly sampled from the full dataset derived from CoRE MOF but are stratified by MOF framework to ensure that all defective structures are in the same set as the pristine structure from which they are generated. Four out-of-domain (ood) sets are included. The “big” ood set corresponds to MOFs from CoRE with over 500 atoms in their unit cell (testing the ability to generalize to larger structures). The “linker”, “topology” ood sets contain linkers and topologies not included in the training data, selected from MOFs in the ultrastable MOF dataset of Nandy et al.^76^ The “linker and topology” ood set contains MOFs from the ultrastable MOF dataset that contain both unseen linkers and topologies. The number of MOF structures and DFT calculations in each set is provided in Table 1, and a more detailed breakdown based on adsorbate type is provided in Table S1. Figure 2 illustrates this detailed distribution across adsorbate types split by task. Further details are described in the Methods section.

**Table 1:** Overview of ODAC23 Dataset Organized by Dataset Split, Number of MOF Frameworks, and Number of DFT Calculations

Split	# pristine MOFs	# defective MOFs	# total MOFs	# total DFT relaxations	# total DFT single points
train	4,537	3,287	7,824	162,224	35,871,295
val	121	71	192	3,998	839,565
test-id	120	93	213	4,669	973,515
test-ood (big)	66	19	85	1,768	381,219
test-ood (linker)	28	0	28	1,182	287,125
test-ood (topology)	55	0	55	1,612	472,256
test-ood (linker and topology)	15	0	15	579	158,773
total	**4,942**	**3,470**	**8,412**	**176,032**	**38,983,748**

### Identification of Selective CO_2_ Adsorption Sites

We used our DFT calculations to directly search for MOFs that are potentially interesting for DAC following the criteria suggested by Findley and Sholl^12^ that the adsorption energy of CO_2_ is < −0.5 eV (with our sign convention, more negative binding energies correspond to more favorable binding) and that the adsorption energy of CO_2_ needs to be more favorable than that of H_2_O. Materials not satisfying the first criterion are unlikely to bind sufficient quantities of CO_2_ at the dilute concentrations relevant for DAC, and materials not satisfying the second criterion are likely to adsorb far more water from air than CO_2_. In the following analysis, we compared the lowest adsorption energy of all computed configurations for each MOF + adsorbate case. We neglected cases with |*E*_ads_/(*n*_CO_2__ + *n*_H_2_O_)| > 2 eV because we suspect these cases are unphysical.

**Figure 3:**
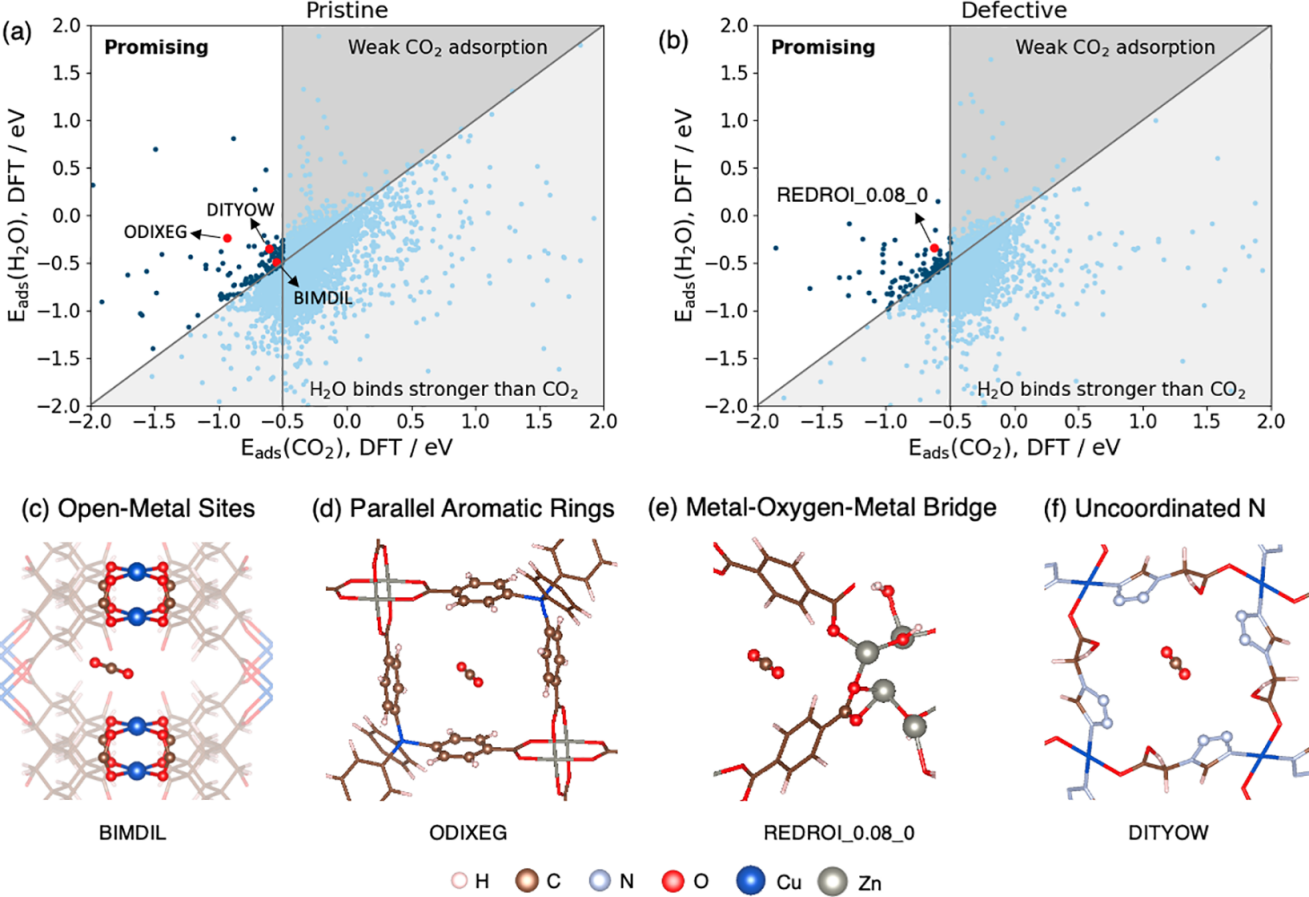
Parity plots showing DFT-calculated CO_2_ and H_2_O adsorption energies in (a) pristine and (b) defective MOFs. (c–f) MOF examples with common features of the promising MOFs.

Figure 3a and b compare the CO_2_ and H_2_O adsorption energies in each pristine and defective MOF from our DFT calculations. As expected, most of the MOFs bind water more favorably than CO_2_. However, 135 of the 5,079 pristine MOFs bind CO_2_ strongly and have higher affinity for CO_2_ than for H_2_O. The top 10 pristine MOFs identified by our DFT calculations with the highest values of |*E*_ads_(CO_2_) – *E*_ads_(H_2_O)| are tabulated in Table S3.

Several screenings of the CoRE MOF database for CO_2_ capture in the presence of water have been conducted previously.^87,88^ Here, we compare our promising MOFs with two previous studies where the adsorption energies of CO_2_ and H_2_O in CoRE MOFs are available. Findley and Sholl performed a similar screening of CoRE MOFs using FF methods, finding no cases that satisfied the criteria stated above.^12^ The observation that our DFT calculations of analogous quantities identified many interesting materials suggests that the generic FFs used previously are insufficiently accurate. Kancharlapall and Snurr recently screened the CoRE MOF 2019 database with a combination of FF and DFT calculations, using somewhat different selection criteria.^89^ Kancharlapall and Snurr also found that FF-based calculations failed to identify MOFs that satisfy our criteria. They further analyzed a subset of their most promising structures using DFT, with a slightly different workflow than we use for ODAC23. We find that 17 materials identified by Kancharlapall and Snurr also appear in the ODAC23 dataset, although we find that 7 of these materials bind H_2_O more strongly than CO_2_ and the remaining 10 MOFs bind CO_2_ weakly (*E*_ads_(CO_2_) ≥ −0.5 eV), indicating that they may not be promising for DAC.

In addition to considering the adsorption of single CO_2_ and H_2_O molecules, we also used DFT to probe the coadsorption of CO_2_ and H_2_O in MOFs. With the resulting coadsorption energies, we computed the adsorbate–adsorbate interaction energies associated with removing both molecules from the coadsorbed state, denoted , for each MOF using eq 6. For the 10 MOFs listed in Table S3, there are three distinct scenarios for this quantity. In a simple case like ZIDBEV,  eV is small relative to the single molecule adsorption energies, so coadsorption can be approximated in a simple way as separate adsorption of the two molecules. For MOFs with negative adsorbate–adsorbate interaction energies like IMAGAG ( eV), coadsorption of CO_2_ and H_2_O is strongly favored relative to adsorption of the individual molecules. Positive adsorbate–adsorbate interaction values such as those seen for IPIDUH ( eV) and TUGTAR ( eV) indicate the coadsorption is much less favorable than adsorption of isolated molecules. In some cases the first adsorbate–adsorbate interaction energies are strongly nonzero (e.g., KOQLUZ,  eV), suggesting that rearrangement of the MOF structure occurred in the coadsorbed case that was not observed for the individual adsorbed molecules.

For the CO_2_ + 2H_2_O configurations, we also computed the second adsorbate–adsorbate interaction energy using eq 7. This energy is small or negative for all of the 10 promising MOFs listed in Table S3. One example, LEWZET, shows an extremely negative second adsorbate–adsorbate interaction energy of −5.48 eV; this occurs because of significant distortion in the relaxed MOF that occurs due to adsorption of a second water molecule. We note that these effects cannot be explored in existing FF-based searches of MOFs, which assume that the MOF structure is unperturbed by adsorbates. It would be challenging, however, to draw in depth conclusions about a selection of MOFs from a limited number of DFT calculations. The complexities associated with the changes in MOF frameworks during coadsorption and the challenges with sampling the many possible placements of coadsorbed states both point to the need to be able to derive FFs or ML models that allow rapid assessment of large numbers of states to provide a thorough description of coadsorption.

Our results also include the first large collection of adsorbed molecules in defective MOFs relaxed with DFT. The cell volume of most of the MOFs decreased after introducing defects (Figure S2a). From the 3,628 defective MOFs, we found 107 defective MOFs with CO_2_ adsorption energy greater than water (Figure 3b). The top 10 defective MOFs ranked in the same way as the pristine materials are listed in Table S4. Defects play an important role in the adsorption of water and CO_2_. For example, pristine TIDLID has an adsorption energy of −1.10 eV for CO_2_ and −0.52 eV for H_2_O (Figure S2b), but defective TIDLID was no longer considered promising because the porous structure collapsed, and the PLD was smaller than 3.3 Å (Figure S2c).

**Figure 4:**
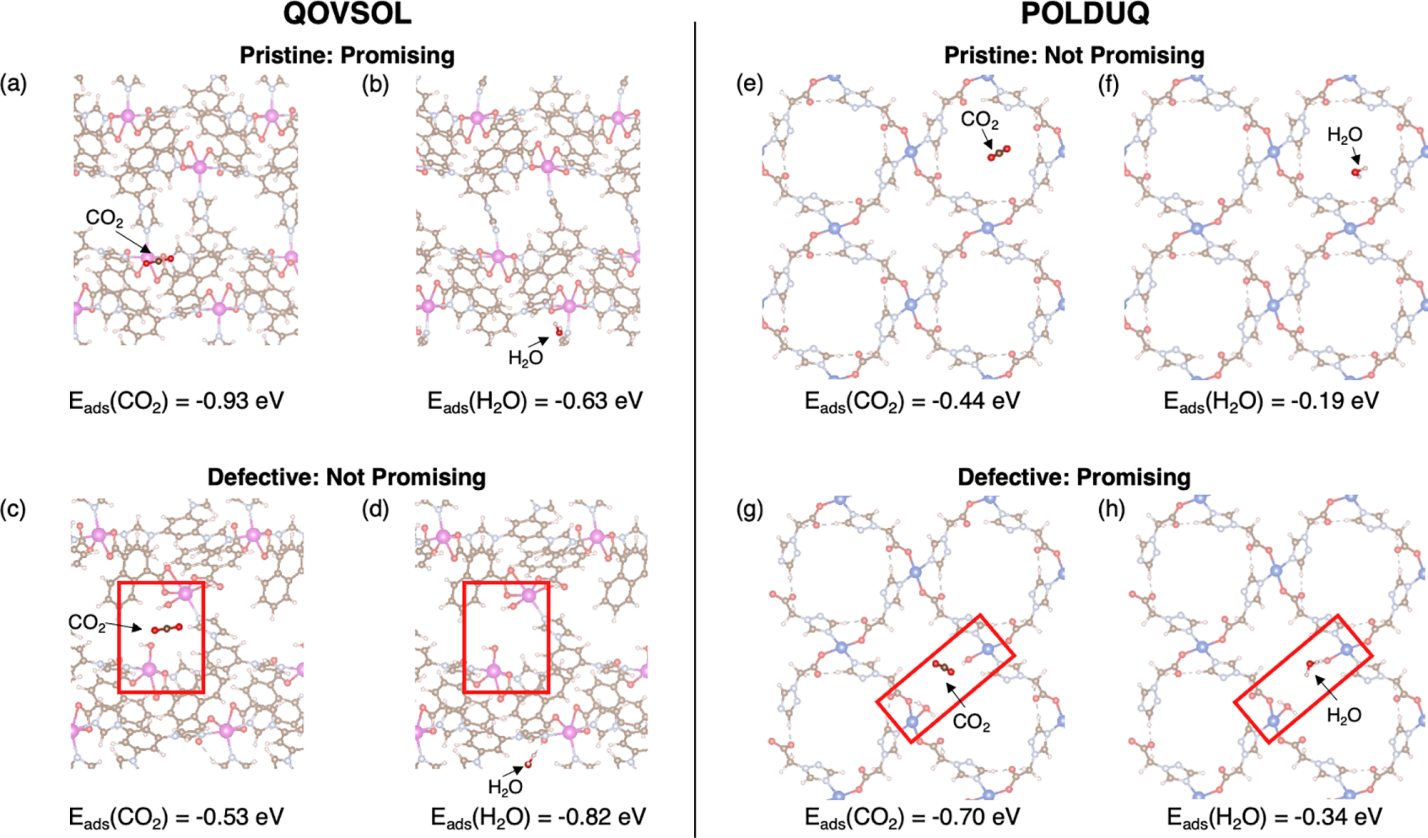
Examples showing different impacts of the defects in MOFs. The defects generated are shown in red squares. Negative impact of defects on DAC (a–d): Defective QOVSOL with a defect concentration of 0.12 shows less favorable CO_2_ adsorption (a and c) and stronger H_2_O adsorption (b and d). Positive impact of defects on DAC (e–g): The H_2_O adsorption is slightly more favorable in defective POLDUQ with a defect concentration of 0.06 (f and h), but the CO_2_ adsorption is much stronger at the defect site (e and g).

The defect concentration was not strongly correlated with the difference in adsorption energies associated with the presence of defects (Figure S3). The average differences of CO_2_ adsorption energy were nearly zero for all defect concentrations, and adding defects to MOFs resulted in slightly more favorable water adsorption on average. However, the effect of defects on adsorption energies differs greatly from case to case. In Figure 4a–d, defects in QOVSOL resulted in more favorable H_2_O adsorption and less favorable CO_2_ adsorption, making it no longer a promising candidate for DAC. On the other hand, our calculations with defective MOFs show that the defects in some of these materials can create interesting adsorption environments for DAC. We found multiple cases where pristine MOFs would not be selected based on the criteria defined above but where the defective material is a promising candidate. Figure 4e–h shows one example of POLDUQ. Our observations are broadly consistent with previous experimental and simulation results for CO_2_ adsorption in UiO-66,^90,91^ and enhanced CO_2_ adsorption in Cu-BTC is due to water coordinated to OMS.^92^ Although defects are capped with water or hydroxyl groups in most cases, it is also possible for defects to create OMSs. The diversity of possibilities illustrates the need for accurate and efficient methods to rapidly explore the many configurations and effects that can exist in defective MOF structures.

It is interesting to ask what motifs or attributes give MOFs adsorption energies that are favorable for DAC. Previous research has suggested several characteristics of good candidates for this application. Boyd et al. identified three favorable characteristics: parallel aromatic rings with a spacing of approximately 7 Å, metal–oxygen–metal bridges, and open-metal sites.^11^ The presence of uncoordinated N atoms or amine groups has also been proposed as a contributing factor to strong CO_2_ adsorption.^93,94^ We examined these four characteristics (Figure 3c–f) in our list of promising MOFs: 224 of the 242 promising MOFs can be characterized by at least one of these characteristics. In particular, 72% of the promising pristine MOFs had open metal sites, 60% had parallel aromatic rings, and slightly under 30% had metal–oxygen–metal bridges or undercoordinated N atoms. For defective MOFs, open metal sites were slightly less prevalent (54%), while the other motifs occurred with similar frequencies (59% parallel aromatic rings, 34% metal–oxygen–metal bridges, and 26% undercoordinated N atoms). To understand whether these features are truly predictive, it is important to compare these results to the prevalence of these features in the total population of pristine/defective MOFs in our data set. In this sense, undercoordinated N atoms are the strongest predictor of strong and selective CO_2_ adsorption for pristine MOFs, being over-represented by 30% in the promising MOF population. Open metal sites are also slightly over-represented by 10% in the promising MOF population. However, these characteristics are neither over- or under-represented in defective MOFs. Parallel aromatic rings are under-represented by 13% in promising pristine MOFs and 15% in promising defective MOFs, while metal–oxygen–metal bridges are under-represented by 21% (pristine) or 31% (defective). Many readers will suspect that amine groups can play an important role in creating materials with a strong affinity for CO_2_. The ODAC23 dataset contains 217 pristine and 254 defective MOFs with an amine functional group. Of these, 7 MOFs (2 pristine and 5 defective) were found to be promising, so amine functional groups were significantly under-represented among promising MOFs. Details of the frequency normalization calculation can be found in Table S5. These findings suggest that open metal sites and undercoordinated N atoms are the chemical motifs that are most suggestive of strong and selective CO_2_ adsorption in pristine MOFs, while none of the previously identified motifs are strong indicators for defective MOFs. This suggests that additional development of ideas characterizing the environments enabling strong and selective CO_2_ adsorption will be useful in the future. Structure files of the promising MOFs and the code for promising MOF analysis are available in our open-source repository on GitHub.^c^

Although the structures in the CoRE MOF database set were derived from experiments, it is important to be cautious in concluding that every structure in this dataset is in fact a real material. In developing the CoRE MOF 2019 database, automatic cleaning procedures were applied to experimentally reported crystal structures, including the removal of solvent molecules and the resolution of partial occupancies. Although this procedure was generally effective, there are cases where it was too aggressive. We observed removal and incorrect partial occupancies in a number of the MOFs listed above. Charge-balancing ions were also removed for MOFs denoted “charged” in Table S4. For each MOF listed above, we manually compared the MOF structures retrieved from the CoRE MOF 2019 database and the original publications. From this analysis, we curated a selection of promising MOFs that are completely charge neutral and where the CoRE MOF structure is fully consistent with the original experimental data. On the basis of current DFT data and manual analysis, we expect these to be the most promising MOFs for experimental synthesis and testing. These MOFs are listed in Tables 2 and 3. The tables include the number of times the original synthesis report has been cited, since this has been suggested as a proxy for the ease of synthesis/reuse of a material, and the tables indicate which of the four promising MOF characteristics mentioned above appear in each material. Two of the ten proposed MOFs have common names: PCN-516 for ODIXEG and PCN-46 for LUYHAP. A complete lookup table for common names available for our list of promising MOFs can be found in Table S6. The available CO_2_ adsorption isotherms from experimental measurements of these MOFs show relatively strong CO_2_ adsorption at low partial pressures,^108^ which is consistent with the implications of our calculations.

**Table 2:** Five Pristine MOFs Suitable for Synthesis on the Basis of ODAC23 Calculations and Manual Evaluation of Original Synthesis Reports

						characteristics				exp. CO_2_ loading (mmol/g)			
MOF	*E*_ads_(CO_2_)	*E*_ads_(H_2_O)	PLD	LCD	Metal	OMS	PAR	M–O–M	Uncoordinated N	150 mbar	1 bar	common name	# of citations
ODIXEG	–0.94	–0.24	7.80	10.4	Zn	*√*	*√*					PCN-516	56^95^
QOVSOL	–0.93	–0.63	3.67	6.21	Cd		*√*		*√*	0.1 (298 K)	0.2 (298 K)^96^		35^97^
QEFNAQ	–0.57	–0.32	4.72	6.03	Cu	*√*	*√*			0.4 (293 K)	1.0 (293 K)^98^		272^99^
FECXES	–0.64	–0.39	6.59	10.83	Cu	*√*	*√*			1.6 (273 K)	6.3 (273 K)^100^		56^100^
DITYOW	–0.60	–0.36	4.79	4.86	Cu	*√*			*√*				52^101^

**Table 3:** Five Defective MOFs Suitable for Synthesis on the Basis of ODAC23 Calculations and Manual Evaluation of Original Synthesis Reports

							characteristics				exp. CO_2_ loading (mmol/g)			
MOF	defect conc.	*E*_ads_(CO_2_)	*E*_ads_(H_2_O)	PLD	LCD	Metal	OMS	PAR	M–O–M	uncoordinated N	150 mbar	1 bar	common name	# of citations
POLDUQ	0.06	–0.70	–0.36	5.09	5.27	Cu	*√*			*√*				12^102^
CUGVUW	0.16	–1.14	–0.82	3.41	5.64	Cu		*√*		*√*				24^103^
PEPKOL	0.08	–0.62	–0.35	3.46	3.92	Ni		*√*						444^104^
SUJNUH	0.12	–0.93	–0.68	6.62	7.08	Cu		*√*			1.3 (195 K)	2.2 (195 K)^105^		77^105^
LUYHAP	0.16	–0.58	–0.37	8.39	12.35	Cu	*√*					3.1 (298 K),^106^ 2.5 (296 K),^107^ 5.2 (270 K)^107^	PCN-46	158^106^

## Evaluation of the Accuracy of Classical Force Fields

Our large library of DFT calculations allowed us to further investigate the accuracy of existing classical FFs against our DFT calculations. We focus here on the energy of interaction between adsorbed molecules and MOFs, since this is the key calculation underlying previous high throughput assessments of MOFs for CO_2_ adsorption. Specifically, we considered a “standard” FF for adsorption in MOFs that combines the UFF4MOF,^109−111^ TraPPE,^112^ and SPC/E^113^ FFs for atoms in the MOF, CO_2_, and H_2_O, respectively. Coulombic interactions were defined using DDEC point charges assigned to MOF atoms from our DFT calculations.^114^ Further technical details are provided in the Methods section.

We computed the interaction energy for 51,478 DFT-relaxed MOF + adsorbate systems using the FF and DFT. These are analogous to the energies in the S2EF task. Using interaction energies for this comparison rather than adsorption energies is consistent with previous FF-based studies that assume framework rigidity.^14,28,115,116^ The ODAC23 dataset also includes information on MOF deformation associated with the presence of adsorbates, and future work could explore how accurately existing FFs for MOF atoms describe these effects.

**Figure 5:**
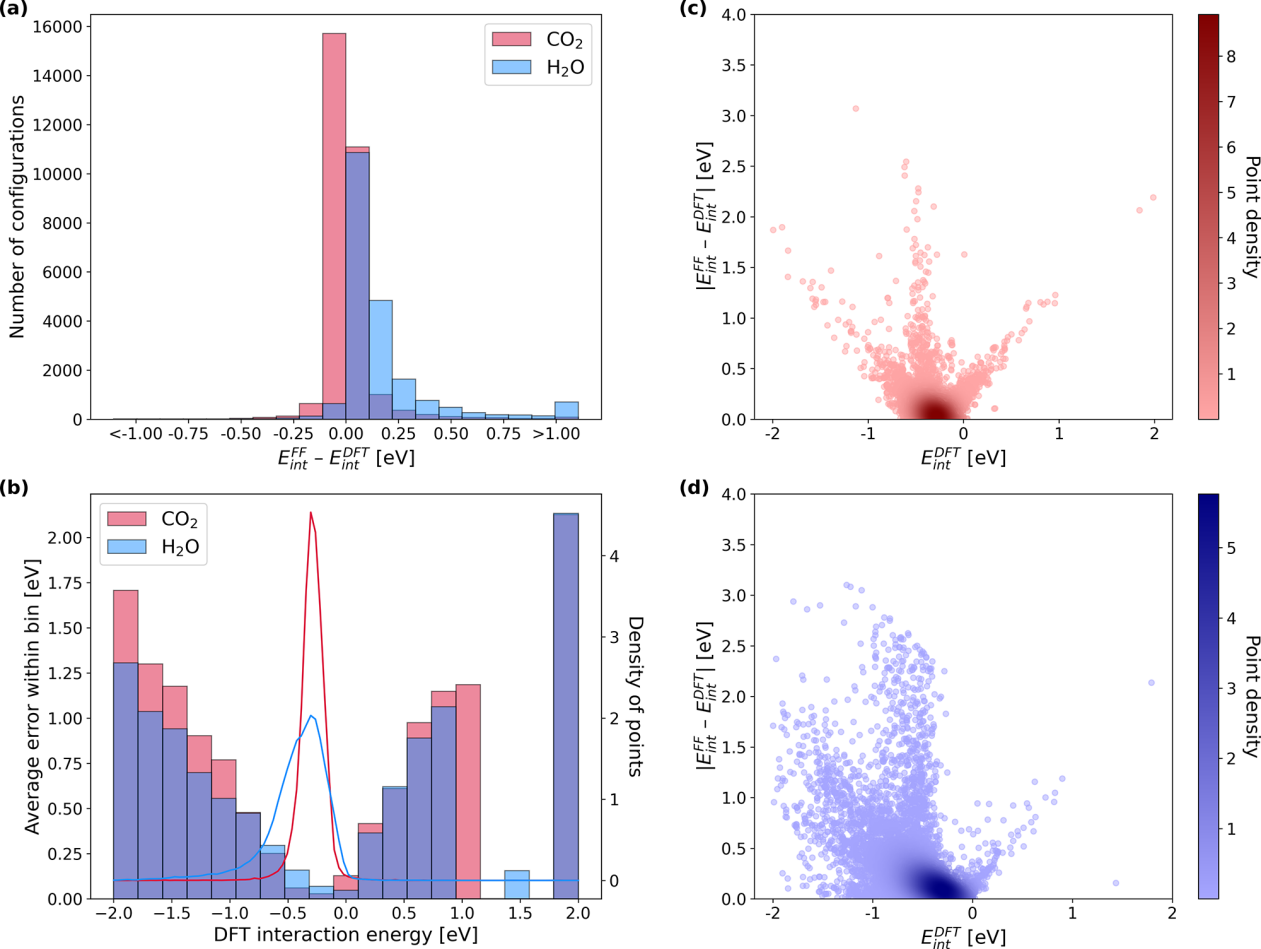
Comparison of adsorbate interaction energies calculated with FFs and DFT. (a) Histogram of energy differences between FF and DFT for 29,644 CO_2_ calculations (red) and 20,892 H_2_O calculations (blue). (b) Binned errors and DFT interaction energy distributions split by adsorbate. (c, d) Absolute difference between FF and DFT energies plotted versus DFT interaction energy for CO_2_ and H_2_O, respectively.

The results of our FF calculations and comparisons to DFT interaction energies are shown in Figure 5. All structures in this comparison contained only one adsorbate molecule (either CO_2_ or H_2_O), and we omit 226 structures with DFT interaction energies outside the range of [−2, 2] eV since we suspect these structures are unphysical. We also omit 716 structures with reasonable DFT energies because their FF predictions also fall outside of [−2, 2] eV. This is done to avoid heavily skewing the subsequent discussion and is revisited at the end of this analysis. Figure 5a shows that in many cases the difference between the classical FF and DFT is less than 0.25 eV and that many of the DFT results can be described as physisorption. van der Waals (vdW) interactions dominate within the physisorption regime of −0.5 ≤ *E*_int_^DFT^ ≤ 0 eV, and if interaction energies are restricted to this range then the mean absolute error (MAE, or simply *error*) between FF and DFT energies is 0.06 eV. This indicates that the physics-based FFs we tested are quite well adapted to predict the interaction energy when physisorption is dominant.

The results in Figure 5b–d provide a less promising view of the classical FF. The error between the FF and DFT calculations scales approximately linearly with the DFT energy outside the physisorption regime, showing that the FF predicts a physisorption energy even when DFT indicates that chemisorption is occurring. The minima in these graphs around −0.4 eV again indicate that the FF is only capable of accurately predicting physisorption. In the chemisorption regime from −2 to −0.5 eV, the MAEs for CO_2_ and H_2_O are 0.29 and 0.39 eV, respectively. Figure 5b shows the number of points and average error as a function of DFT interaction energy. Although relatively few points outside the physisorption regime exist, the FF interaction energy errors increase drastically with the magnitude of the interaction energy. Many interesting chemistries that are beneficial for DAC occur due to chemisorption (e.g., CO_2_ binding more strongly than H_2_O). These cases would be missed by a classical FF that is unable to model chemisorption. There are also many instances in which the FF energy prediction is substantially larger than the DFT-calculated energy. We attribute these to cases involving chemisorption where the adsorbate is close to the framework and therefore returns very large Lennard-Jones energies. That is, the FF exhibits unstable behavior here because very slight changes in geometry cause large spikes in energy predictions.

An additional takeaway from Figure 5 is that H_2_O is significantly more challenging to model than CO_2_. This is consistent with the fact that physics-based water models are complex and are themselves the subject of a rich body of literature.^117^ We found that the error in interaction energy calculations within the [−2, 2] eV domain involving H_2_O (0.19 eV) was more than triple for CO_2_ (0.05 eV). The vast majority of unstable FF calculations involved H_2_O and not CO_2_. Selecting and implementing an appropriate water model is a nontrivial task that further complicates the use of classical FFs for material screening.

Finally, there are a number of cases where the FFs predict very large interaction energies, with the maximum error being 187.2 eV. These cases typically correspond to dissociative adsorption, where the FF is not an appropriate model. Figure S4 presents the binned FF errors as a function of the DFT interaction energy for all configurations with a DFT interaction energy in [−2, 2] eV, irrespective of whether the FF interaction energy falls within this range. Comparison with Figure 5b shows that the 716 cases with reasonable DFT energies but unreasonable FF energies drastically increase the error, and that catastrophic failures (e.g., errors >10 eV) begin to dominate when the DFT adsorption energies are stronger than 1 eV. The large errors cause the FF MAE for all structures to be quite large at 0.28 eV. If the MAE is calculated only for cases where the FF interaction energy is in the range of [−2, 2] eV, then the classical FF performs reasonably well with an interaction energy MAE of 0.11 eV across 50,536 calculations. Overall, the results indicate that the FF performs well for physisorption but fails to capture strong chemical interactions that are likely critical for DAC.

### Training and Analysis of Machine Learning Models

We begin by training and benchmarking models for the S2EF task, since it is the most general. We tested six graph neural network (GNN) architectures for this task: SchNet,^118^ DimeNet+,^119,144^ PaiNN,^120^ GemNet-OC,^121^ eSCN,^122^ and EquiformerV2.^123^ We chose models that performed well on the OC20 and OC22 benchmarks since those datasets and tasks are most similar to ours. These models use GNNs containing equivariant or nonequivariant operations to compute energies and forces. All models were trained to minimize the following objective function for forces and energies:
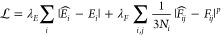
2where the loss coefficients λ_*E*_ and λ_*F*_ are used to trade-off the force and energy losses. *E*_*i*_ and  are, respectively, the ground truth and predicted energies of system *i*, and *F*_*ij*_ and  are, respectively, the ground truth and predicted forces for the *j*th atom in system *i*. The number of atoms in system *i* is denoted by *N*_*i*_. *p* is the order of the norm; SchNet and DimeNet++ used *p* = 1, while the other models used *p* = 2.

We used the same model sizes as those used for OC20 (Table S7). To prevent overfitting due to the smaller size of the data set, we adjusted the weight decay for each model. We also slightly adjusted the initial learning rates, batch sizes, learning rate schedules, and the loss coefficients λ_*E*_ and λ_*F*_. All error metrics are reported for test sets that were not included in the training and optimization process. Additional information can be found in the Methods section.

**Figure 6:**
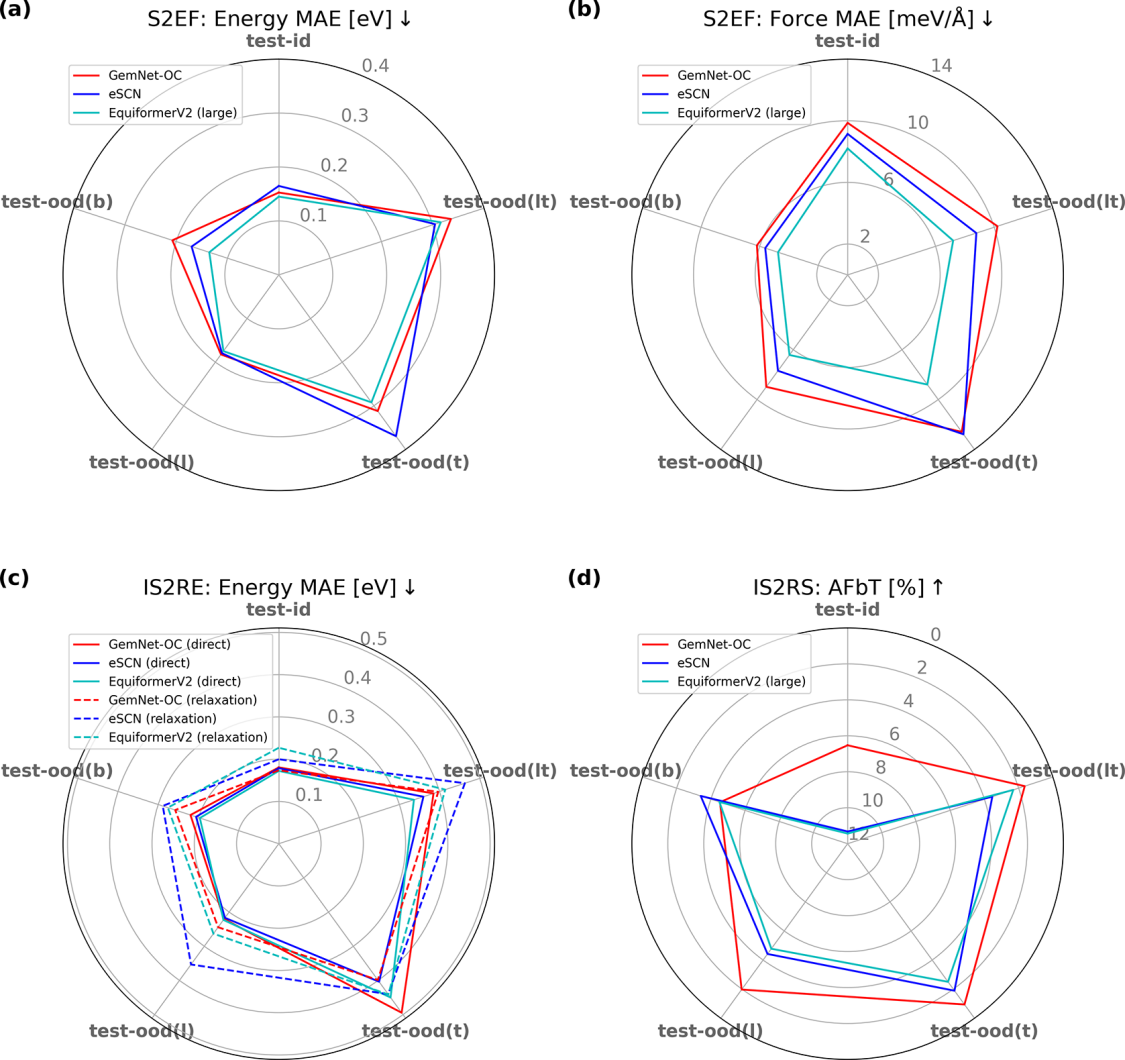
Radar plots for S2EF (a) energy and (b) force MAEs, (c) IS2RE energy MAEs, and (d) IS2RS AFbT for the top three best models—GemNet-OC (red), eSCN (blue), and EquiformerV2 (large, except in (c) where the lighter model is shown) (cyan). Dashed lines correspond to the relaxation approach for IS2RE; all other models are direct predictions. Axes correspond to different in- and out-of-domain test sets and are aligned so that the best result is closest to the origin of the plot in all cases.

The results of all ML models on the S2EF task are presented in Table S8, revealing that GemNet-OC, eSCN, and EquiformerV2 have the best performance. Figure 6 shows a radar plot comparing these models, indicating that EquiformerV2 (large) achieved the best results for both forces and energies, with a force MAE of 8.20 meV/Å and energy MAE of 0.15 eV on the in-domain test set. The eSCN and GemNet-OC models also performed well, with force MAEs of less than 10 meV/Å and energy MAEs of under 0.17 eV. The models’ relative performance was consistent with their performance on the OC20 and OC22 datasets, suggesting that improvements in model architecture generalize to various materials datasets.

Next, we consider how the models generalize to out-of-domain test sets. The results in Table S8 and Figure 6 demonstrate that the EquiformerV2 (large) model outperforms the other models on most metrics for all out-of-domain sets. The ML models show only a slight decrease in performance on the test-ood(b) and test-ood(l) sets, suggesting that they generalize well to larger graphs or to new linker chemistry. However, the energy predictions for the test-ood(t) and test-ood(lt) sets are substantially worse than the test-id set, although the force errors are similar to the other test sets. This could be due to errors in long-range vdW interactions for unseen topologies, since this is the main contribution that varies with topology.

**Figure 7:**
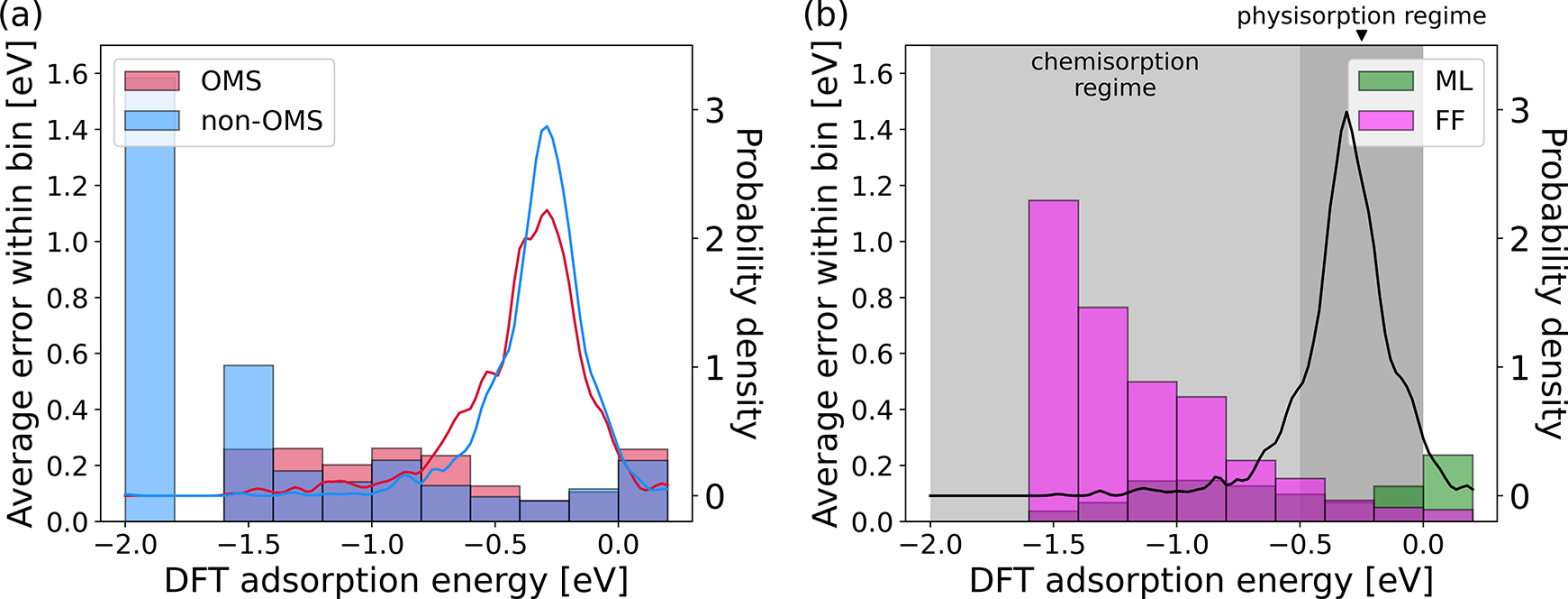
Binned errors and relative density of the number of points (solid lines) as a function of DFT adsorption energy for (a) ML predicted adsorption energies on open metal site (OMS) (red) and non-OMS (blue) and (b) interaction energies predicted by FFs (magenta) and corresponding adsorption energies predicted by ML (green) models. Compared to FFs, ML models are significantly more accurate in the chemisorption regime and are comparable in the physisorption regime. Positive adsorption energies are omitted from the plot because they are rare and likely unphysical; plots with the full range of adsorption energies are provided in Figure S4.

We also analyze the performance of the models on the more complex chemical environments of OMSs and defects. OMSs are significant for DAC as they can enable stronger CO_2_ adsorption.^46^ Classical FFs are known to be less accurate for MOFs with OMSs as they can cause high polarization in adsorbed molecules.^46,124^Tables S9 and S10 compare GemNet-OC, eSCN, and EquiformerV2 on different subsets of the test-id split. Table S9 shows the performance across pristine MOFs with and without OMSs, and Table S10 compares the performance of the same models on pristine and defective structures. The ML models have similar force MAEs on the OMS and non-OMS sets, as well as the pristine and defective sets. However, the energy MAEs are lower for MOFs without OMSs or defects. This may be due to the stronger and more complex interactions at OMSs or may be related to the relative abundance of different types of examples within the dataset. Figure 7a analyzes the binned error for MOFs with and without OMSs, indicating that errors are slightly higher for OMS-containing MOFs in the chemisorption regime, suggesting that the ML models perform slightly worse at predicting the more complex chemical interactions at OMS sites.

A direct comparison between classical FFs and ML models is not feasible because the architecture of the FFs makes it challenging to relax framework atoms. However, we can compare the S2EF adsorption energy errors to the interaction energy errors from FFs to gain insight, since both evaluate the ability to describe interactions between frameworks and adsorbates. We did this with 1,391 relaxed single-adsorbate configurations in the test-id set, which is a subset of the 50,536 structures that excludes all systems used in ML model training. For this reason, energy errors reported in this section may vary slightly from those in the evaluation of the accuracy of classical force fields. The energy MAE for EquiformerV2 (large) for these systems was 0.10 eV, while the MAE for the FF interaction energies on the same structures was 0.49 eV. It is clear that, on average, the best ML models outperform the classical FF models, even when only focusing on relaxed single-adsorbate geometries. However, a more detailed analysis reveals that the large FF error occurs due to a small number of large failures. The maximum force field error is 67.66 eV, compared to a maximum error of 1.23 eV for the EquiformerV2 (large) model. If the analysis is restricted to the cases where force fields predict interaction energies in the range of [−2, 2] eV, the average errors are quite comparable, with MAEs of 0.10 eV for both.

In the regime where adsorption energies range from −0.5 to 0 eV and physisorption is expected to be dominant, the FF performance becomes comparable to that of ML, with an MAE of 0.10 eV for the FFs and 0.09 eV for the ML models. A detailed analysis is provided in Figure 7b, which indicates that ML models exhibit consistently lower errors in the chemisorption regime, in contrast to FF models, which fail for chemisorption. Given the importance of chemisorption in selective CO_2_ capture at low concentrations, this finding supports the potential value for ML models for DAC. See Figure S5 for errors in the repulsive region. We note that our FF calculations used “general purpose” FFs that are readily suitable for high throughput calculations. Systematic methods exist to improve classical FFs by including parameters derived from first-principle calculations,^44^ and the data sets we have introduced may create useful opportunities to develop improved FFs using variations of these methods.

Next, we move to the IS2RE and IS2RS tasks, which evaluate the ability of ML models to directly predict the relaxed adsorption energy (IS2RE) and structure (IS2RS) from an initial guess of framework and adsorbate positions. The IS2RE task only predicts energy and is evaluated with the energy MAE (similar to S2EF) and the “energy within threshold” (EwT), which evaluates the fraction of predictions within 0.02 eV of the DFT energy. The IS2RE task can be solved by training ML models to directly predict the relaxed adsorption energy from the initial structure (the *direct* method), or by running a structure relaxation with an S2EF model (the *relaxation* method). In the case of the relaxation approach, the task is identical to IS2RS, where the energy of the final structure is used as the IS2RE prediction. However, the metrics used to evaluate the IS2RS task are significantly different, since the goal is to compare structures. The metrics used are the average distance within threshold (ADwT), force below threshold (FbT), and average force below threshold (AFbT), with details provided in the Methods. Evaluating the IS2RS models is quite expensive since it requires performing a DFT single-point for each of the predicted relaxed structures. Therefore, we only evaluated the best 4 models (GemNet-OC, eSCN, EquiformerV2, and EquiformerV2 (large)) and only computed DFT single-point energies on 500 randomly selected structures from each test split.

For the IS2RE task, any S2EF model can be used for the indirect approach, so we evaluated all six S2EF models from this work by performing structure relaxations with each model. The resulting structures are also used for the IS2RS task. In addition, we selected the best three models—GemNet-OC, eSCN, and EquiformerV2—and retrained them for the direct approach, with settings identical to the corresponding S2EF models unless otherwise noted.

Figure 6 and Table S11 show the results for the IS2RE task on each of the test splits. On the test-id set, the direct methods obtain an energy MAE around 0.18 eV and an EwT of over 10%. The relaxation approach with older S2EF models like SchNet, DimeNet++, and PaiNN performs worse than direct methods, while newer methods such as GemNet-OC, eSCN, EquiformerV2, and EquiformerV2 (large) are marginally better than direct approaches. Similar to the S2EF task, we find that the performance of the ML models degrades marginally on the test-ood(b) or test-ood(l) datasets, while they degrade significantly on the test-ood(t) and test-ood(lt) datasets. This is true for both direct and relaxation-based approaches.

Figure 6 and Table S12 show the IS2RS results on each test split. The ADwT results are reasonably high for the test-id and test-ood(b) sets but degrade significantly for test-ood(l) and test-ood(t) sets. However, the results on the DFT-based metrics (FbT and AFbT) indicate that the models achieve relaxed structures consistent with what would be obtained from DFT < 1% of the time in all cases (and 0% in many cases). This inconsistency between ADwT and (A)FbT has also been observed for OC20^75^ and indicates that the models need significant improvement to achieve the level of accuracy needed to replace DFT for the prediction of relaxed structures. However, the fact that the models are able to predict the energies of relaxed structures with reasonable accuracy in the IS2RE task is an encouraging sign, since the state of the art for high throughput MOF screening with force field is to assume that the structures are rigid. This assumption becomes particularly questionable in the case of defective MOFs or strong adsorption, indicating the need for models capable of accounting for relaxation effects.

**Figure 8:**
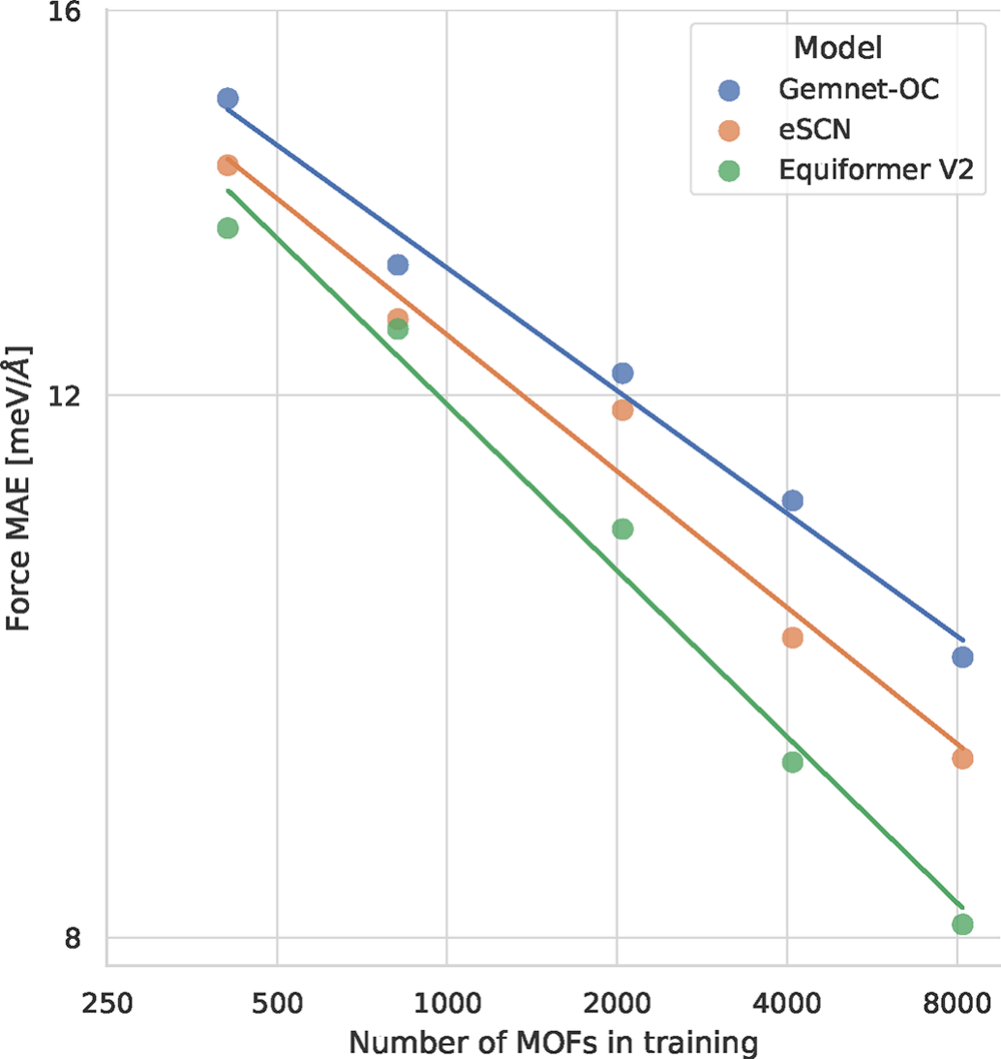
Force MAE on the test-id set for the top 3 S2EF models when trained on different amounts of training data. The lines show scaling laws obtained by fitting a line between log of the force MAE and log of the number of training MOFs for each model.

It is clear that the ML models presented here demonstrate significant promise compared to the standard classical FF models. However, there are also obvious deficiencies. One advantage of ML models is that they tend to improve with more data. In particular, scaling laws for deep learning models relate model performance to a parameter like the number of model parameters or size of the training dataset. Scaling laws have helped to choose the optimal model and training parameters in several domains.^125−127^Figure 8 shows the scaling laws for the ODAC23 dataset size, comparing the force MAEs of different models as a function of the number of MOFs in the training data. Consistent with previous work in other domains, we observe a power-law relationship between force MAE and the number of MOFs. This implies that we can continue to improve the performance of these models by including more training data. It is also interesting to note that equivariant models like EquiformerV2 and eSCN have better scaling properties than GemNet-OC, matching the findings of Batzner et al.^128^ This indicates that the use of more sophisticated model architectures is a promising route forward.

Based on these scaling laws, a much larger number of MOFs would be required to achieve force MAEs of 3 meV/Å (approaching the numerical error of DFT). An alternative strategy common in deep learning is to leverage similar datasets. This has proven useful in the Open Catalyst Project models,^129^ and we plan to explore this approach in future work. Another possible strategy is to develop model architectures that are tailored for the DAC application. In particular, the strong performance of FFs in the weak-binding regime suggests that incorporating information on vdW interactions into the model^130^ or Δ-ML^131^ models may be promising strategies. Ultimately, we expect that improved model architectures, advanced transfer learning, and joint training techniques may provide a route to leveraging physical knowledge and other large atomistic datasets to improve performance on ODAC23, although we leave this as future work.^132,133^

## Impact and Future Outlook

The results of this study provide the most comprehensive DFT dataset of CO_2_ and H_2_O adsorption in MOFs available to date. Analysis of the resulting DFT calculations has shown that, contrary to the findings from FF-based studies, there are numerous MOF-based adsorption sites with strong and selective CO_2_ adsorption. A direct comparison of the DFT results to classical FFs provides the most comprehensive perspective to date on the accuracy of FFs. The results reveal that the FFs work well in cases where vdW interactions dominate but fail when stronger bonding is involved. These findings demonstrate that high-throughput screening with methods capable of treating chemisorption and framework distortion will be required to identify MOFs that can strongly and selectively bind CO_2_ under humid conditions.

In addition, the work provides a benchmark for state-of-the-art ML models for CO_2_ and H_2_O adsorption in MOFs. The results indicate that the best performing GNN models, such as EquiformerV2, are capable of predicting adsorption energies with average errors of ∼0.15–0.3 eV and forces with errors of ∼5–10 meV/Å. Comparison with classical FFs shows that these ML models are more accurate outside the regime of vdW interactions. This, coupled with the importance of strong binding in identifying selective CO_2_ adsorption sites, suggests that these ML models have the potential to replace classical FFs as the standard approach in high-throughput MOF screening for DAC and other applications in separations and catalysis.

Moving forward, it will be important to critically evaluate and improve ML models and associated datasets so that they can be applied to other steps in the computational sorbent selection process. For example, grand canonical Monte Carlo simulations are critical for predicting adsorption isotherms. The models here are untested for this task since they have not seen configurations with more than two adsorbed molecules. It would be very interesting to test predictions of these models against DFT data from higher loadings generated with methods that can sample the full range of possible configurations such as *ab initio* MD. This is especially critical for the case of bicomponent CO_2_/H_2_O isotherms that are needed to predict the behavior of MOF materials in DAC process models. The complex mixture of vdW interactions, hydrogen bonding, and covalent bonding in H_2_O makes it difficult to accurately predict these bicomponent isotherms with existing methods, but the ML models presented here provide a promising foundation for future developments.

## Methods

### ODAC23 Dataset Generation

A workflow diagram with details on the dataset generation workflow is provided in Figure S6, and more details are provided in the subsections below.

#### Structure Relaxations

DFT relaxations used the PBE exchange–correlation functional^81^ with a D3 dispersion correction^82^ including Becke-Johnson damping and with spin polarization.^83^ Relaxations were performed with conjugate gradient methods with a step size of 0.01, and Gaussian smearing was used with a width of 0.2 eV. A plane wave cutoff energy of 600 eV to minimize effects of Pulay stress and a precision of 10^–5^ eV were used. All simulations were performed in the Vienna Ab Initio Simulation Package (VASP) v5 software with a 1 × 1 × 1 k-point grid.^134^

We relaxed all 8,803 CoRE MOF pristine structures using DFT as described above before generating defective structures and placing adsorbate molecules, and a total of 5,079 MOFs converged. DFT convergence failures are due to a variety of issues. For example, Chen and Manz identified several failure modes in CoRE MOF input files beyond overlapping atoms (3.5% of all screened structures), including isolated atoms (7.8%), misbonded hydrogens (1.3%), and over-/underbonded carbons (15.3%).^79^ Examples of VASP convergence issues were large systems that took too long or ran out of memory (∼10% of screened structures) and numerical errors pertaining to Hamiltonian diagonalization. We noticed several converged structures with very high initial formation energies (>3 eV/atom). All initial inputs of converged structures were thus screened for overlapping atoms resulting from imperfect solvent removal processes and partial occupancies in the CoRE work. We used the published list of effective atomic radii by Chen and Manz for atom typing; a structure failed if any atom pairs were less than half the sum of their respective atomic radii apart.^79^ In total, 161 structures failed and were excluded from further analysis due to overlapping atoms and unphysically large initial formation energies.

#### Defective MOF Generation

We expanded the pristine set of MOFs from CoRE MOF by introducing missing linker defects using the methods introduced recently by Yu et al.^85^ This approach requires identification of the linker and nodes in each MOF, a task completed using the algorithm MOFid developed by Bucior et al.^135^ Out of 5,079 pristine MOFs that converged in our DFT calculations, we successfully identified the nodes and linkers of 4,780 MOFs. In each MOF, we created structures with different defect concentrations from 0.01 to 0.16, where the defect concentration is defined as the number of removed linkers divided by the total number of linkers. For MOFs that have multiple types of linkers, we generated corresponding defective structures by removing one kind of linker at a time. OMSs were capped using either a water molecule if the removed linker is charge neutral or hydroxyl(s) if the removed linker was charged to create structures that have no overall charge. In total, 16,358 distinct structures were generated and relaxed by DFT, and 6,340 of them converged. We kept only the relaxed structures with PLD > 3.3 Å, and the final set of defective MOFs contained 3,470 frameworks.

#### Adsorbate Placement

In each relaxed MOF (either pristine or defective) structure, we placed an adsorbate(s) using nonbonded pairwise interactions defined by one of the classical FFs by the RASPA 2.0 package.^33^ FF parameters for framework atoms and adsorbates (CO_2_ and H_2_O) were defined by the United Force Field (UFF)^109^ and TraPPE-United Atom FF,^136^ respectively. Specifically, we adopted the rigid TIP5P model for H_2_O molecules.^137,138^ The Lorentz–Berthelot mixing rules and a tail correction with a cutoff radius of 14 Å were used to define the Lennard–Jones interactions between MOFs and adsorbates. Coulombic interactions were considered when partial charges of the framework atoms were available by the DDEC method. We collected configurations of [MOF + CO_2_] or [MOF + H_2_O] from every 10,000 Monte Carlo cycles with the same translation, rotation, and reinsertion probabilities. We took two approaches to ensure that structures do not have duplicated positions and exhibit diversity in structures: (i) energy matching and (ii) random sampling. The energy matching approach notes that different nonbonded interaction energies will correspond to different configurations. Starting from the minimum observed energy, we sampled configurations in 5 kJ/mol intervals until the nonbonded interaction energy reached a threshold (−15 kJ/mol and −5 kJ/mol for CO_2_ and H_2_O, respectively). If the minimum energy was greater than the threshold, we included only the configuration with the minimum energy. No configuration was added for cases where the minimum energy was >0 kJ/mol. This resulted in having 0–9 adsorbate placements for each MOF structure, leading to a diverse collection of more than 10,000 MOF + adsorbate configurations per adsorbate by the energy matching approach. For random sampling, we randomly chose 2 configurations from the collection of 10,000 cycles and added these configurations to the set selected from energy matching, leading to more than 16,000 MOF + adsorbate configurations per adsorbate by the random sampling approach. Several MOF structures were further excluded from the dataset because their pore size shrunk during the relaxation, making it impossible for RASPA to place an adsorbate in their pores. We manually added 158 converged [MOF + H_2_O] configurations to position water molecules closer to OMSs. This was done for MOF structures where a CO_2_ molecule was near OMSs without nearby water or when they were identified as promising but with fewer than 4 H_2_O placements.

In addition to considering the adsorption of single CO_2_ and H_2_O molecules, we also used DFT to probe the coadsorption of CO_2_ and H_2_O in MOFs. Similar to our calculations with single adsorbed molecules, these calculations allowed for full relaxation of the adsorbate and MOF degrees of freedom. Coadsorption studies include the following examples: [1CO_2_ + 1H_2_O] and [1CO_2_ + 2H_2_O]. In each study, we inserted all of the participating molecules into each empty MOF structure. Since we are interested in the behavior of CO_2_ in the presence of water, we discarded configurations where the distance between the centers of mass for any pair of adsorbate molecules was greater than 5 Å. For MOF structures whose primitive cells were too small to place multiple adsorbates in the pores, the primitive cell was repeated to form a bigger supercell. Whether a supercell was used to save the configuration can be found on GitHub.^d^ Both energy matching and random sampling strategies were applied to multi-adsorbate configurations. The energy threshold was set to be −5 kJ/mol for all molecule combinations in the case of the energy matching approach.

After placing adsorbates, we performed DFT structure relaxations on each MOF + adsorbate configuration. We used the same DFT settings as the MOF relaxations but with fixed unit cell parameters.

#### Out-of-Domain MOF Selection

The ODAC23 dataset contains four out-of-domain (OOD) test sets in addition to the in-domain test set to evaluate the ability of ML models trained on the ODAC23 dataset to new topologies, new linker chemistries, and larger MOFs.

The **test-ood (big)** or test-ood(b) test split only contains MOFs with over 500 atoms in their unit cells. Testing on this set allows us to assess how well our models generalize to larger MOFs than those contained in the training set.

The other three OOD test sets were designed to study how our ML models generalize to new chemistries and topologies not present in CoRE MOF. To create these splits, we sampled structures from the “ultrastable MOF database” developed by Nandy et al.^76^ To create our OOD test sets, we selected the ultrastable MOFs with less than 500 atoms and contained either novel linkers or topologies not present in the rest of our dataset. This allowed us to create three OOD test sets: the **test-ood (linker)** set contains novel linkers but known topologies, the **test-ood (topology)** set contains novel topologies but known linkers, and the **test-ood (linker & topology)** set contains both novel linkers and novel topologies. We abbreviate these three sets as test-ood(l), test-ood(t), and test-ood(lt) respectively. We used the MOFid library^135^ to identify the organic linkers and topologies.

We believe that the inclusion of these OOD sets, which are biased to a property not related to the DAC application, provides a useful test of the generalizability of our trained ML models.

### Energy Definitions

We defined three energy definitions for analysis of our work. Throughout this section, *E*_A_^B^ denotes the total energy of a system of interest A calculated by a method B. If not specifically noted, B defaults to DFT. Energies are a function of atomic coordinates (C) either from DFT relaxation (*r*_C_^relax^) or from a single-point DFT calculation (*r*_C_^single^).

### Adsorption Energy

The adsorption energy can be defined as
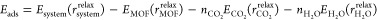
3where *E*_system_ is the DFT energy of the MOF + adsorbate system, *E*_MOF_ is the reference DFT energy of the relaxed standalone MOF, *n*_CO_2__ and  denote the number of CO_2_ and H_2_O molecules in the system respectively, and *E*_CO_2__ and  are the gas phase energies of the corresponding molecules.

In eq 3, the structure of the MOF in the system and the standalone MOF are from separate DFT relaxations. When a supercell was created during adsorbate placement, the reference energy *E*_MOF_ was computed by performing an additional DFT relaxation on the supercell without the adsorbate.

The inclusion of adsorbate molecules during relaxation broke framework symmetry and resulted in lower energy empty MOF configurations in a small number of cases. We conducted a second round of relaxations on these empty MOFs and successfully found lower energy states for 690 pristine and 625 defective MOFs. These lower energy states were used as the reference energy for all adsorption energy calculations. We removed all configurations where the adsorption energy was found to be < −2 eV per adsorbate.

We also define  for which we obtained the total energy of the current MOF + adsorbate configuration instead of seeking its relaxed state. This can be expressed as
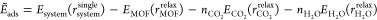
4 indicates how far the current state of a MOF + adsorbate system is from its reference state and is used as one of the main targets in our ML studies. In the case that  is computed from the single-point DFT calculation of a relaxed structure (i.e., *r*_system_^single^=*r*_system_^relax^), it is equivalent to *E*_ads_.

### Interaction Energy

The interaction energy is defined as
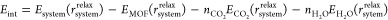
5where *E*_int_ was calculated either by DFT (*E*_int_^DFT^) or the classical FF (*E*_int_^FF^).

Interaction energy calculations were performed only on the relaxed MOF + adsorbate configurations using single-point DFT. For simplicity, interaction energies were computed only in single adsorption cases, and thus *n*_CO_2__ + *n*_H_2_O_ = 1 in eq 5.

### Adsorbate–Adsorbate Interaction Energy

The adsorbate–adsorbate interaction energy quantifies interactions between adsorbates in coadsorption cases and is defined as
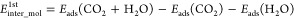
6
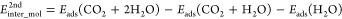
7where the number of each adsorbate is shown in parentheses. The first adsorbate–adsorbate interaction energy  shows the adsorbate–adsorbate interactions between CO_2_ and H_2_O, and the second adsorbate–adsorbate interaction energy  shows the adsorbate–adsorbate interactions induced by introducing a second H_2_O molecule.

### Evaluation Metrics

For all machine learning models, we used the same evaluation metrics used for OC20. We briefly describe the metrics used for each task in this section, but refer the reader to the OC20 paper^75^ for more details.

### Structure to Total Energy and Forces (S2EF)

The S2EF task is evaluated on the accuracy of force and adsorption energy predictions through the following metrics. For these metrics,  is computed by eq 4.

• Energy MAE: Mean absolute error between the predicted energy and the ground truth DFT energy:
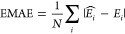
8where *E*_*i*_ and  are the ground truth and predicted energies of system *i* and *N* is the total number of systems.

• Force MAE: Mean absolute error between predicted and ground truth DFT forces:
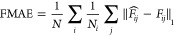
9where *F*_*ij*_ and  are the predicted and ground truth forces on the *j*-th atom of system *i* and *N*_*i*_ is the number of atoms in system *i*.

- Force Cos: Cosine similarity between the predicted and ground truth forces.
- Energy and forces within the threshold (EFwT): The fraction of energies and forces that are respectively within 0.02 and 0.03 eV/Å of the ground truth DFT values.

### Initial Structure to Relaxed Energy (IS2RE)

The IS2RE task is evaluated on the accuracy of relaxed energy predictions using the following metrics. For these metrics, *E* ≡ *E*_ads_ is computed by eq 3.

- Energy MAE: Mean absolute error between predicted energy and the ground truth DFT energy of the relaxed state.
- Energy within Threshold (EwT): The fraction of energies within 0.02 eV of the DFT relaxed energy.

### Initial Structure to Relaxed Structure (IS2RS)

The IS2RS task is evaluated on whether the predicted relaxed structure is close to a local minimum in the energy landscape using the following metrics.

- Average Distance within Threshold (ADwT): Distance within Threshold (DwT) is the percentage of structures with an atom position MAE below a threshold β. ADwT averages DwT across thresholds ranging from β_0_ = 0.01 Å to β_1_ = 0.5 Å in increments of 0.001 Å.
- Force below Threshold (FbT): Percentage of relaxed structures with maximum DFT calculated per-atom force magnitudes below a threshold of α = 50 meV/Å. This is only computed for structures that satisfy the DwT criterion with β = 0.5 Å.
- Average Force below Threshold (AFbT): FbT averaged over a range of thresholds: α_0_ = 10 meV/Å to α_1_ = 400 meV/Å in increments of 1 meV/Å.

As the systems in ODAC23 do not contain any fixed atoms, per-atom metrics like Force MAE, ADwT, FbT, and AFbT are computed over all atoms. Note that a new single point DFT calculation is required to evaluate FbT and AFbT on a given data point.

### Classical Force Fields

All classical FF calculations in this work used the readily available MOF extension to the ubiquitous UFF force field (UFF4MOF)^110,111^ in the Large-scale Atomic/Molecular Massively Parallel Simulator (LAMMPS).^36^ Topology files were generated using LAMMPS Interface.^139^ CO_2_ and H_2_O molecules were described using the TraPPE^112^ and SPC/E^113^ models, respectively. The SPC/E model was chosen to avoid challenges related to the geometry of massless sites in newer models such as TIP5P,^137^ which was used for adsorbate placement but can be cumbersome to place in high-throughput FF calculations. Electrostatic interactions were described using DDEC framework point charges provided as part of the ODAC23 dataset,^114^ and long-range interactions were computed using an Ewald summation with a force tolerance of 10^–5^ kcal/mol/Å. The cutoff for all pairwise interactions was 12.5 Å. Periodic boundary conditions were applied in all calculations, and tail corrections were not applied. Code for FF calculations is available in our open-source repository on GitHub.^e^

### ML Models

Various ML FF models have been proposed for molecular and material tasks over the past few years.^118,120−123,140−142^ Here, we benchmark a subset of the state-of-the-art models on our tasks. All of our models were implemented using PyTorch,^143,144^ and the code is available in our open-source repository on GitHub.^f^

For S2EF, we trained SchNet,^118^ DimeNet++,^119^ GemNet-OC,^142^ PaiNN,^140^ eSCN,^122^ and EquiformerV2^123^ models. We trained 2 versions of the EquiformerV2 model—a small 31M parameter model and a large 153M parameter model. The list of models used is summarized in Table S7. Edges were computed on-the-fly using a nearest-neighbor search with a cutoff of 8 Å, a maximum of 50 neighbors for SchNet, DimeNet++, and PaiNN, and a maximum of 20 neighbors for eSCN and EquiformerV2. GemNet-OC uses different cutoffs for different types of interaction triplets and quadruplets. These S2EF models can then be used to run machine learning relaxations to solve the IS2RE and IS2RS tasks. We benchmarked the top performing S2EF models—GemNet-OC, eSCN, and EquiformerV2—to run these ML relaxations using the L-BFGS optimizer for 125 steps or until the magnitude of the predicted forces on each atom was less than 0.05 eV/Å. IS2RE can also be solved by directly predicting the energy from the initial system, which we call *direct IS2RE prediction*. We trained GemNet-OC, eSCN, and EquiformerV2 models on the direct IS2RE task.

### Comparison of Computational Cost

Machine learned potentials involve a much higher number of floating point operations than classical force fields. However, most of these operations involve matrix operations which are well-suited for modern graphics processing units (GPUs). Therefore, ML models can be run efficiently on a GPU in terms of wall clock time. Since these potentials are often used to run structure relaxations, we computed the average wall clock time per relaxation for UFF, GemNet-OC, and EquiformerV2 models across 60 randomly sampled systems from the dataset. We ran the UFF relaxations on a CPU and the ML relaxations on a GPU since those are the most suitable architectures. On average, a UFF relaxation requires 37.67 s per CPU core on a Dual Intel Xeon Gold 6226R 2.9 GHz CPU machine. In comparison, GemNet-OC relaxations take 0.8 s, and EquiformerV2 relaxations take 10.2 s on a single 32GB V100 GPU.

The comparison between the runtimes is not straightforward because of the difference in hardware. For a fair comparison, we estimate the cost of running 1000 relaxations on a public cloud: on AWS, it costs $0.55 with GemNet-OC, $7 with EquiformerV2, and $1.2 with UFF. From these numbers, we conclude that ML models and UFF have a comparable computational cost.

## Data Availability

The ODAC23 data set and all the trained ML models are publicly available at our official Web site (https://open-dac.github.io/) and the open-sourced GitHub repository (https://github.com/Open-Catalyst-Project/ocp).

## Supporting Information Available

The top 20 metal atoms in the data set; examples of defective structures; the impact of defect concentration on adsorption energies; extended version of Figure 7; the workflow detailing the relaxation of MOF structures; additional details on the data set; the top 7 organic linkers in the data set; the top 10 promising pristine and defective MOF structures; a compilation of ML model architectures used in this study, along with their corresponding error metrics for the tasks (PDF)
Transparent Peer Review report available (PDF)

## Footnotes

a https://open-dac.github.io/.

b https://github.com/Open-Catalyst-Project/ocp.

c https://github.com/Open-Catalyst-Project/odac-data/tree/main/promising_mof.

d https://github.com/Open-Catalyst-Project/odac-data/tree/main/supercell_info.csv.

e https://github.com/Open-Catalyst-Project/odac-data/tree/main/force_field.

f https://github.com/Open-Catalyst-Project/ocp.
