# Supplementary material for: The Open DAC 2023 Dataset and Challenges for Sorbent Discovery in Direct Air Capture
Source: ACS Cent Sci. 2024 May 1;10(5):923–41. doi: 10.1021/acscentsci.3c01629 (PMC11117325; doi:10.1021/acscentsci.3c01629)
Supplement: Supplementary file 1 — oc3c01629_si_001.pdf [file oc3c01629_si_001.pdf]

Supporting Information for

The Open DAC 2023 Dataset and Challenges for Sorbent Discovery in Direct Air Capture

Anuroop Sriram,[*,†] Sihoon Choi,[†,‡] Xiaohan Yu,[‡] Logan M. Brabson,[‡] Abhishek Das,[†] Zachary Ulissi,[†] Matt Uyttendaele,[†] Andrew J. Medford,[*,‡] and David S. Sholl[*,‡,¶]

†*Fundamental AI Research, Meta AI, Meta, Menlo Park, CA, USA*
‡*School of Chemical and Biomolecular Engineering, Georgia Institute of Technology, Atlanta, GA, USA*
¶*Oak Ridge National Laboratory, Oak Ridge, TN, USA*

E-mail: anuroops@meta.com; ajm@gatech.edu; shollds@ornl.gov

Supplementary Figures

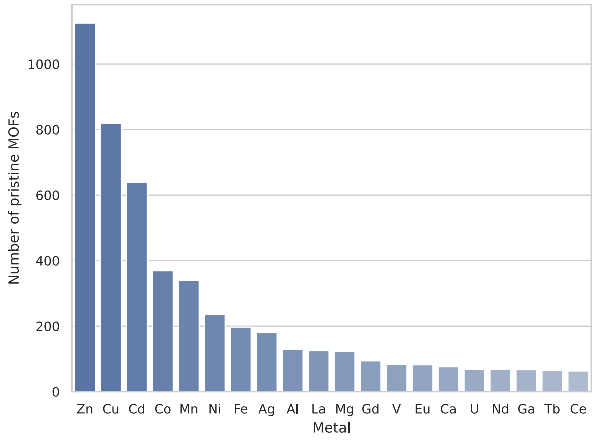

Figure S1: Top 20 metal atoms in pristine MOFs.

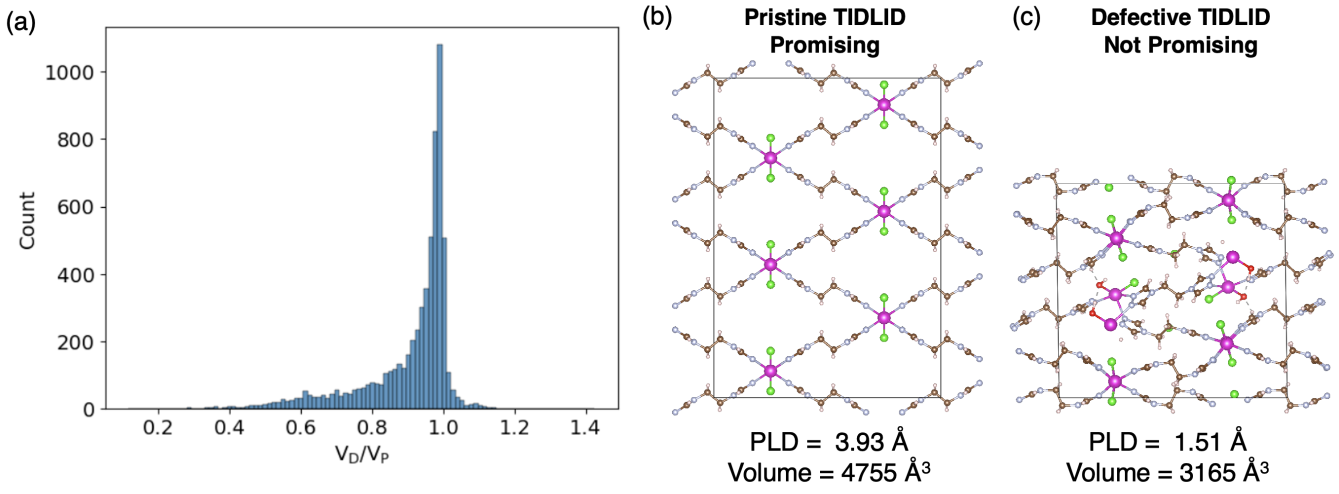

Figure S2: (a) A histogram of the ratio of the defective MOF volume (V_D) over the pristine MOF volume (V_P), with all structures fully DFT relaxed. The structure of relaxed (b) pristine and (c) defective TIDLID with a defect defect concentration of 0.08 , showing structural collapse on defect formation.

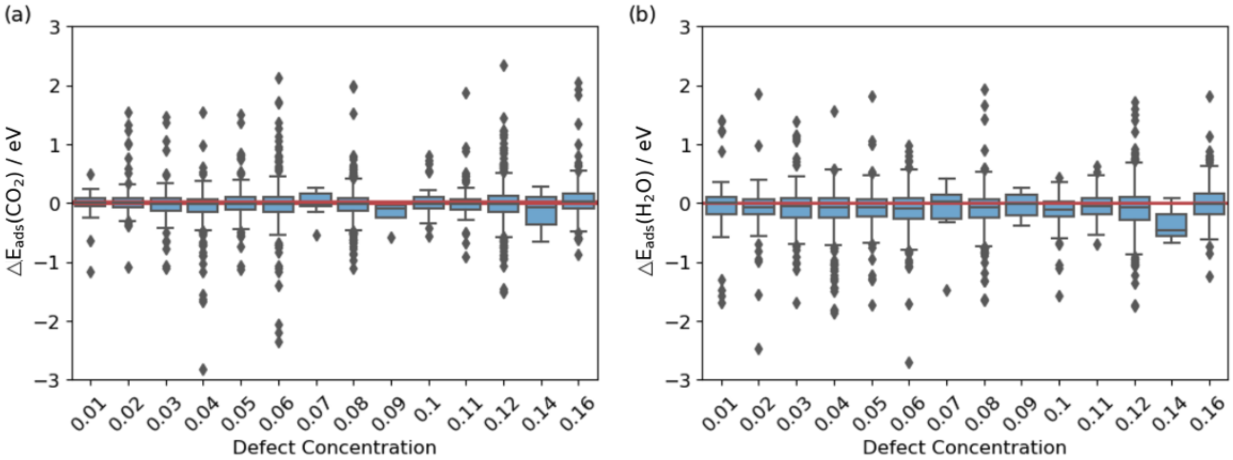

Figure S3: The influence of defect concentration on the adsorption energies of (a) CO_2 and (b) H_2O in MOFs. $\Delta E = E_{\text{Defective}} - E_{\text{Pristine}}$.

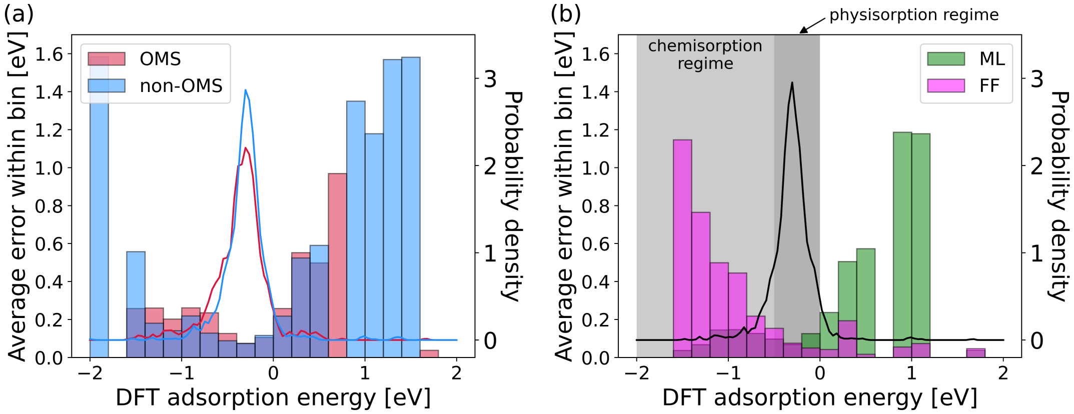

Figure S4: Binned errors and relative density of the number of points (solid lines) as a function of DFT adsorption energy for (a) ML predicted adsorption energies on open metal site (OMS) (red) and non-OMS (blue) and (b) interaction energies predicted by FFs (magenta) and corresponding adsorption energies predicted by ML (green) models. This plot is an extension of Figure 7, displaying DFT adsorption energies within the range of -2 eV to 2 eV.

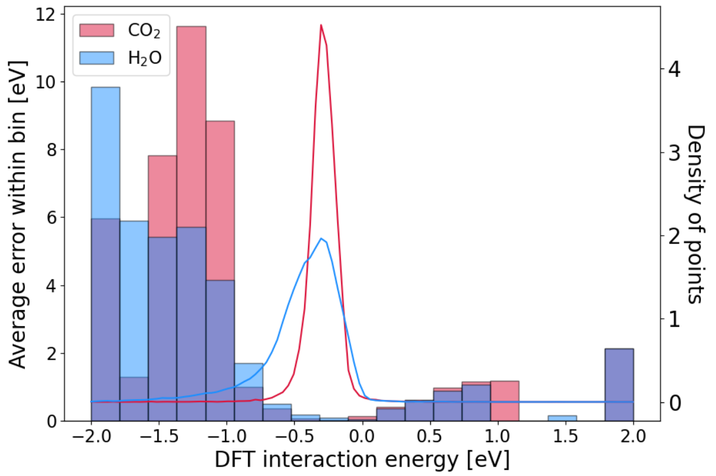

Figure S5: Binned FF errors and DFT interaction energy distributions split by adsorbate for all 51,252 systems with $-2 \leq E_{\mathrm{int}}^{\mathrm{DFT}} \leq 2$ eV irrespective of FF interaction energy.

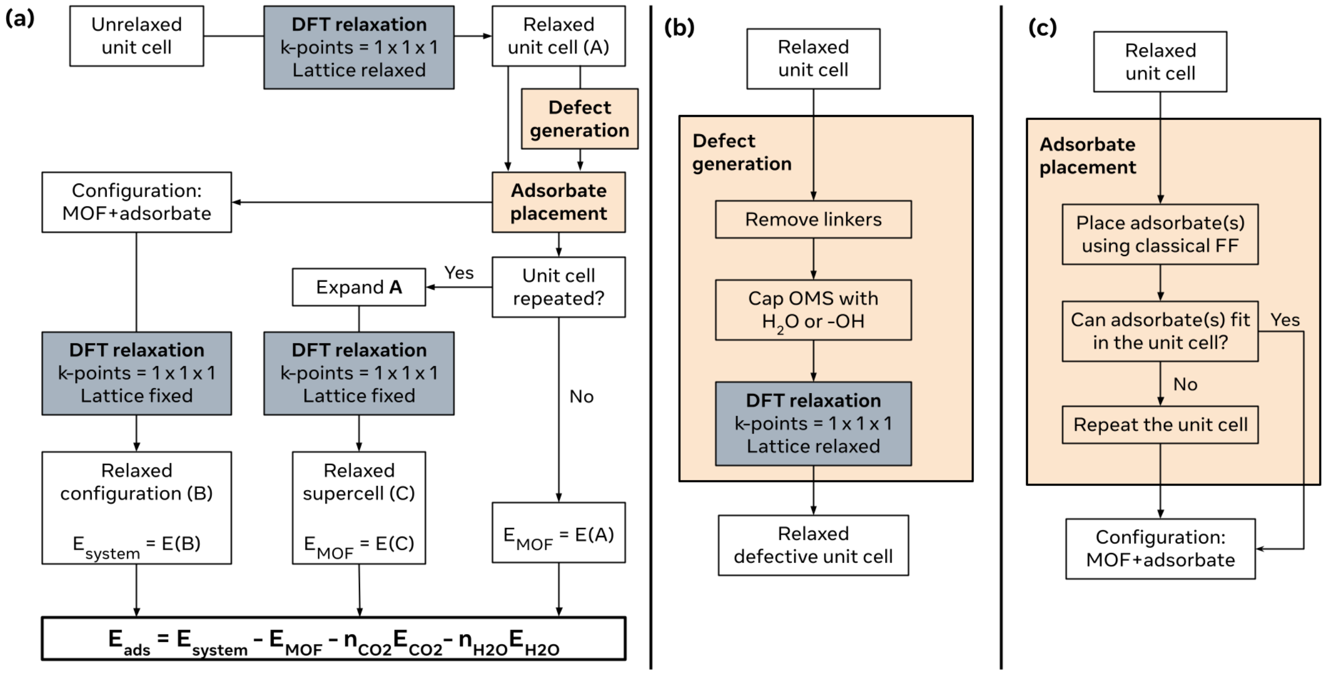

Figure S6: The workflow for generating and relaxing pristine and defective MOF structures in ODAC23.

Supplementary Tables

Table S1: Summary of ODAC23 dataset organised by task and split

Task	Split	MOF + CO_2	MOF + H_2O	MOF + CO_2+ H_2O	MOF + CO_2+ $2H_2O$	Total
	train	6,608,649	5,196,597	13,092,633	10,973,416	35,871,295
	val	160,841	125,984	310,093	242,647	839,565
	test-id	163,574	133,372	360,413	316,156	973,515
S2EF	test-ood (big)	62,718	63,711	136,374	118,416	381,219
	test-ood (linker)	50,392	14,242	99,376	123,115	287,125
	test-ood (topology)	71,384	60,308	182,255	158,309	472,256
	test-ood (linker & topology)	27,281	24,351	60,121	47,020	158,773
	Total	**7,144,839**	**5,618,565**	**14,241,265**	**11,979,079**	**38,983,748**
	train	46,274	34,456	48,373	33,121	162,224
	val	1,138	862	1,211	787	3,998
	test-id	1,291	972	1,420	986	4,669
IS2RE/IS2RS	test-ood (big)	533	383	534	318	1,768
	test-ood (linker)	355	87	383	357	1,182
	test-ood (topology)	439	306	533	334	1,612
	test-ood (linker & topology)	166	135	172	106	579
	Total	**50,196**	**37,201**	**52,626**	**36,009**	**176,032**

Table S2: Top 7 organic linkers in the pristine MOFs

SMILES	Structure	Number of Pristine MOFs
`[O-]C(=O)c1ccc(cc1)C(=O)[O-]`	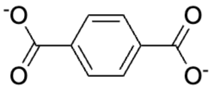	316
`[O-]C(=O)c1cc(cc(c1)C(=O)[O-])C(=O)[O-]`	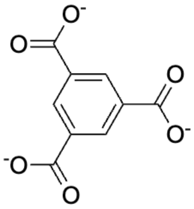	244
`C#N`	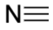	220
`n1ccc(cc1)c1ccncc1`	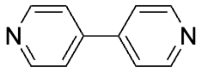	216
`[O-]P(=O)([O-])[O-]`	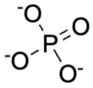	157
`[O-]C(=O)C(=O)[O-]`	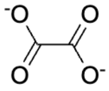	151
`[O-]C=O`	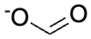	99

Table S3: Top 10 promising pristine MOFs with stronger CO_2 adsorption energy compared to H_2O by DFT calculations [eV]

MOF	$E_{\text{ads}}(CO_2)$	$E_{\text{ads}}(H_2O)$	$E_{\text{ads}}(CO_2)$ - $E_{\text{ads}}(H_2O)\uparrow$	$E_{\text{ads}}(CO_2$+ $H_2O)$	$E^{\text{1st}}_{\text{inter\_mol}}$	$E_{\text{ads}}(CO_2 + 2H_2O)$	$E^{\text{2nd}}_{\text{inter\_mol}}$
IPIDUH	-1.98	0.32	-2.30	-0.63	1.04	-2.51	-2.20
KOQLUZ	-1.50	0.70	-2.19	-3.11	-2.31	-3.54	-1.13
LEWZET	-0.89	0.81	-1.69	0.14	0.23	-4.53	-5.48
TUGTAR	-1.74	-0.47	-1.27	-1.70	0.51		
cm503311x_alf175K	-0.64	0.48	-1.12	-0.03	0.13	-2.20	-2.66
TONWUO	-1.71	-0.63	-1.09	-2.68	-0.34	-3.78	-0.48
IMAGAG	-1.14	-0.07	-1.07	-1.85	-0.64		
EGIFUV	-1.45	-0.41	-1.04	-0.43	1.42	-0.87	-0.03
ZIDBEV	-1.91	-0.91	-1.00	-2.83	0.00	-4.40	-0.66
PETWIW	-0.72	0.28	-0.99	-1.38	-0.93	-1.09	0.01

Table S4: Top 10 promising defective MOFs with stronger CO_2 adsorption energy compared to H_2O by DFT calculations [eV]

MOF	Defect conc.	$E_{\text{ads}}(CO_2)$	$E_{\text{ads}}(H_2O)$	$E_{\text{ads}}(CO_2)$-$E_{\text{ads}}(H_2O)\uparrow$	$E_{\text{ads}}(CO_2$+ $H_2O)$	$E^{\text{1st}}_{\text{inter\_mol}}$	$E_{\text{ads}}(CO_2 + 2H_2O)$	$E^{\text{2nd}}_{\text{inter\_mol}}$
AFENEE	0.12	-1.86	-0.34	-1.52	-0.81	1.40	-1.65	-0.50
OKIXIQ_charged	0.12	-1.29	-0.09	-1.20	-1.09	0.29	-0.86	0.31
IDAGEA_charged	0.06	-1.18	-0.25	-0.93	-1.23	0.21	-2.42	-0.94
PEGCAH	0.06	-1.60	-0.77	-0.83	-2.83	-0.47	-3.13	0.46
COTXEQ	0.12	-0.60	0.15	-0.74	-1.05	-0.59	-1.56	-0.66
ODAHIK_charged	0.12	-1.02	-0.30	-0.71	-1.60	-0.28	-1.97	-0.06
HUWDEJ	0.12	-1.36	-0.65	-0.71	-1.19	0.82	-2.30	-0.46
HAJLUA	0.16	-0.93	-0.26	-0.68	-3.83	-2.64	-1.95	2.14
HAJLOU	0.16	-1.02	-0.35	-0.67	-1.80	-0.43	-4.32	-2.16
MALRUM	0.04	-1.29	-0.65	-0.64	-2.14	-0.20	-2.86	-0.07

Table S5: Motif frequency normalization results in the pristine and defective MOFs

Specificity*	OMS	PAR	M-O-M	Uncoordinated N	Amine
Pristine	1.10	0.87	0.69	1.28	0.34
Defective	0.97	0.85	0.79	1.02	0.63

Specificity$_i$ =(N$_{promising_i}/N_i)/(N_{promising}/N_{total})$. $N_{promising\_i}$ is the number of promising MOFs with motif i. N_i is the total number of MOFs in the database with motif i. $N_{promising}$ is the total number of promising MOFs. N_{total} is the total number of MOFs in the pristine/defective population.

Table S6: Available Common Names for Promising MOFs

Code Name	Common Name
ANUGOG	UMCM-153
CAVNOE	MIL-53(Sc)-NO2
cm501138g_si_002	CuBTC
DEJRUH	BIF-23
DICKEH	NJU-Bai12
EGIFUV	UCR-9
FAGQAI	TCM-10
FEYJOJ	MOF-502
FIZPOV	JUC-118
IXEJOM	PCN-700-(CF3)2
IYEHAX	Cu-PEIP
jp302979a_si_002	USF2-RuBpy
LUDKOM	STAM-1 acetaldehyde
MIXBIG_auto	NTU-113
NASREH	UPC-8
NEVVEQ	CUK-2
ODIXEG	PCN-516
OLOPEM	TMU-5 (Cd 15%)
RUBTAK03_auto	UiO-66
science.1056698_manual	MOF-14
SUSZOW	STA-15
WIKLOT	EDP-U4
WIRMOB	PMOF-1
XALDAS	WUF-10
XAMDUM07	CuBTC
XANMUX04	FJU-53-Br
XITYOP	PCN-14
YILJAG	MAF-X11
ZILFOR_manual	IFMC-16
CODFUX_0.06_0	MAP-GIS1
HIFTOG02_0.08_0	MOF-5
HOWQEQ_0.08_0	JUC-68
IKETOH_manual_0.12_0	CuBTC
KOJZIT_0.16_0	MOF-602
LUYHAP_0.16_0	PCN-46
MAKXAZ_0.16_1	RPM4-Zn
MIMFOF_0.03_1	AlPO-BDA
MIMFOF_0.1_1	AlPO-BDA
MUNDAC_0.12_0	JLU-Liu15
NABMUA_0.16_0	Mg-MOF-1
NIGDEO_0.14_1	PCN-88
NOKJON_0.12_0	UZnP-2
NUNCEG_0.12_0	LiTCS
SADMUH_SL_0.06_0	MIL-53
VANXUD_0.04_0	UCSB-3
VUKROK_0.16_0	DUT-8(Ni)
XADDIR_manual_0.12_0	NENU-29, CuBTC

Table S7: List of ML model architectures used in this work and the different tasks they were used for

List of ODAC23 Models

Model	# of Parameters	Equivariant Reps	Tasks			
			S2EF	IS2RE-direct	IS2RE-Relax	IS2RS
SchNet[118]	9.1M	✗	✓	*	*	*
DimeNet++[144]	1.8M	✗	✓	*	*	*
PaiNN[120]	20.1M	✓	✓	*	*	*
GemNet-OC[121]	38.9M	✗	✓	✓	✓	✓
eSCN[122]	51.6M	✓	✓	✓	✓	✓
EquiformerV2[123]	31.1M	✓	✓	✓	✓	✓
EquiformerV2 (large)[123]	153M	✓	✓	✓	✓	✓

* Skipped because S2EF results were not competitive

Table S8: Results on the S2EF task for the various test splits

Structure to Energy and Forces (*S2EF*) Test

Split	Model	Force MAE [meV/Å] ↓	Force Cos ↑	Energy MAE [eV] ↓	EFwT ↑
test-id	Median baseline	16.02	0.001	0.406	0.00%
	SchNet	14.44	0.254	0.368	0.02%
	DimeNet++	14.31	0.226	0.416	0.02%
	PaiNN	13.04	0.345	0.241	0.11%
	GemNet-OC	9.87	0.605	0.153	1.16%
	eSCN	9.15	0.658	0.165	1.84%
	EquiformerV2	7.26	0.674	0.182	1.97%
	EquiformerV2 (large)	8.20	0.685	0.145	2.61%
test-ood(b)	Median baseline	7.98	0.001	0.334	0.00%
	SchNet	8.16	0.146	0.529	0.00%
	DimeNet++	7.85	0.157	0.728	0.00%
	PaiNN	7.53	0.242	0.282	0.12%
	GemNet-OC	6.19	0.495	0.207	0.81%
	eSCN	5.62	0.559	0.170	1.15%
	EquiformerV2	4.91	0.610	0.166	1.92%
	EquiformerV2 (large)	4.75	0.612	0.135	3.07%
test-ood(l)	Median baseline	14.65	0.000	0.378	0.00%
	Schnet	13.36	0.262	0.474	0.00%
	Dimenet++	12.15	0.253	0.501	0.01%
	PaiNN	11.47	0.378	0.252	0.05%
	Gemnet-OC	8.98	0.640	0.182	0.29%
	eSCN	7.69	0.719	0.179	0.59%
	EquiformerV2	6.85	0.760	0.161	1.36%
	EquiformerV2 (Large)	6.42	0.761	0.175	2.03%
test-ood(t)	Median baseline	16.18	0.000	0.677	0.00%
	SchNet	14.83	0.181	1.001	0.00%
	DimeNet++	13.62	0.183	1.297	0.00%
	PaiNN	18.18	0.267	0.507	0.01%
	GemNet-OC	12.59	0.488	0.312	0.05%
	eSCN	12.79	0.560	0.370	0.09%
	EquiformerV2	10.19	0.617	0.341	0.52%
	EquiformerV2 (large)	8.80	0.631	0.292	0.51%
test-ood(lt)	Median baseline	13.71	0.000	0.528	0.00%
	SchNet	13.23	0.234	0.746	0.00%
	DimeNet++	12.44	0.234	0.886	0.00%
	PaiNN	11.97	0.349	0.417	0.00%
	GemNet-OC	10.22	0.589	0.335	0.05%
	eSCN	8.78	0.680	0.305	0.25%
	EquiformerV2	7.31	0.727	0.302	0.52%
	EquiformerV2 (large)	7.20	0.720	0.316	0.48%

Table S9: Comparison of S2EF metrics for MOFs with and without OMSs.

S2EF Test - Open Metal Sites

Model	OMS		Non-OMS	
	Energy MAE [eV] ↓	Force MAE [meV/Å] ↓	Energy MAE [eV] ↓	Force MAE [meV/Å] ↓
Median baseline	0.433	16.25	0.355	15.50
GemNet-OC	0.164	10.04	0.129	9.47
eSCN	0.186	9.29	0.120	8.82
EquiformerV2	0.204	8.18	0.169	6.85

Table S10: Comparison of S2EF metrics for pristine and defective MOFs.

S2EF Test - Pristine vs Defective

Model	Pristine		Defective	
	Energy MAE [eV] ↓	Force MAE [meV/Å] ↓	Energy MAE [eV] ↓	Force MAE [meV/Å] ↓
Median baseline	0.406	16.02	0.395	12.12
GemNet-OC	0.153	9.87	0.182	8.05
eSCN	0.165	9.15	0.199	7.36
EquiformerV2	0.187	8.11	0.176	6.58

Table S11: Full results of metrics for IS2RE task on all data splits.

Initial Structure to Relaxed Energy (*IS2RE*) Test				
Split	Method	Model	Energy MAE [eV] ↓	EwT ↑
test-id	Direct	GemNet-OC	0.181	10.40%
		eSCN	0.179	11.11%
		EquiformerV2	0.172	10.77%
	Relaxation	SchNet	0.485	3.10%
		DimeNet++	0.496	3.23%
		PaiNN	0.225	9.10%
		GemNet-OC	0.174	12.18%
		eSCN	0.200	12.33%
		EquiformerV2	0.227	11.59%
		EquiformerV2 (large)	0.169	14.47%
test-ood(b)	Direct	GemNet-OC	0.220	7.13%
		eSCN	0.206	8.31%
		EquiformerV2	0.197	7.35%
	Relaxation	SchNet	0.621	1.64%
		DimeNet++	0.801	1.36%
		PaiNN	0.238	6.73%
		GemNet-OC	0.258	7.43%
		eSCN	0.289	9.54%
		EquiformerV2	0.276	6.96%
		EquiformerV2 (large)	0.179	8.98%
test-ood(l)	Direct	GemNet-OC	0.220	8.84%
		eSCN	0.217	12.50%
		EquiformerV2	0.223	12.50%
	Relaxation	Schnet	0.621	3.72%
		Dimenet++	0.651	2.20%
		PaiNN	0.248	7.01%
		GemNet-OC	0.244	8.19%
		eSCN	0.353	5.32%
		EquiformerV2	0.241	9.98%
		EquiformerV2 (large)	0.232	10.47%
test-ood(t)	Direct	GemNet-OC	0.494	4.05%
		eSCN	0.404	4.73%
		EquiformerV2	0.450	8.11%
	Relaxation	SchNet	1.040	0.62%
		DimeNet++	1.327	0.43%
		PaiNN	0.473	5.14%
		GemNet-OC	0.399	8.04%
		eSCN	0.440	7.23%
		EquiformerV2	0.441	6.39%
		EquiformerV2 (large)	0.366	8.15%
test-ood(lt)	Direct	GemNet-OC	0.385	6.68%
		eSCN	0.360	7.02%
		EquiformerV2	0.336	6.51%
	Relaxation	SchNet	0.711	2.05%
		DimeNet++	1.116	0.68%
		PaiNN	0.410	5.14%
		GemNet-OC	0.397	8.45%
		eSCN	0.463	5.10%
		EquiformerV2	0.414	6.56%
		EquiformerV2 (large)	0.405	9.87%

Table S12: Full results of metrics for IS2RS task on all data splits.

Initial Structure to Relaxed Structure (*IS2RS*) Test				
Split	Model	ADwT ↑	FbT ↑	AFbT ↑
test-id	GemNet-OC	85.46%	0.00%	6.53%
	eSCN	85.13%	0.40%	11.32%
	EquiformerV2	87.92%	0.00%	12.05%
	EquiformerV2 (large)	87.37%	0.60%	11.44%
test-ood(b)	GemNet-OC	87.79%	0.00%	4.54%
	eSCN	86.30%	0.60%	3.41%
	EquiformerV2	88.57%	0.00%	3.28%
	EquiformerV2 (large)	87.50%	0.40%	4.50%
test-ood(l)	GemNet-OC	69.74%	0.00%	1.97%
	eSCN	66.56%	0.40%	4.42%
	EquiformerV2	74.34%	0.00%	4.83%
	EquiformerV2 (large)	75.44%	0.00%	4.78%
test-ood(t)	GemNet-OC	60.03%	0.00%	0.95%
	eSCN	60.08%	0.00%	1.89%
	EquiformerV2	68.23%	0.00%	1.58%
	EquiformerV2 (large)	66.97%	0.20%	2.50%
test-ood(lt)	GemNet-OC	59.11%	0.00%	1.64%
	eSCN	61.27%	0.00%	3.54%
	EquiformerV2	68.30%	0.00%	3.27%
	EquiformerV2 (large)	65.58%	0.20%	2.32%
