# Supplementary material for: The Open DAC 2023 Dataset and Challenges for Sorbent Discovery in Direct Air Capture
Source: ACS Cent Sci. 2024 May 1;10(5):923–41. doi: 10.1021/acscentsci.3c01629 (PMC11117325; doi:10.1021/acscentsci.3c01629)
Supplement: Supplementary file 2 — oc3c01629_si_002.pdf [file oc3c01629_si_002.pdf]

oc-2023-01629m.R1

Name: Peer Review Information for "The Open DAC 2023 Dataset and Challenges for Sorbent Discovery in Direct Air Capture"

First Round of Reviewer Comments

Reviewer: 1

Comments to the Author

Sholl and co-workers explore a new ML-driven approach to the discovery of MOF structures interesting for DAC application. They further generate and present a new dataset containing DFT calculations on >8000 MOFs. The amount of work put into this manuscript is very high and the results are very interesting both from the point of view of materials discovery and method optimization. The paper should be considered for publication, however, I have several suggestions from the MOF point of view:

1) The authors talk about the concept of open metal sites (OMS) in MOFs and study their impact on water/CO2 (co-)adsorption. Since contradicting definitions of OMS exist across the literature, it would be important for the authors to define OMS and explain in detail which criteria are used to decide on an MOF having or not having an OMS. As such, the authors also mention that such sites are often "capped" with water, hydroxyl groups or – in reality – solvent molecules left from the synthesis. Does it for example mean that a Ti-O-H moiety is an open metal site?

Following this, OMS can appear as a result of linker-removal (in defect-containing MOFs) OR can also be a part of the general, undefective structure. Do you think you can provide further insight into the difference between these two classes of MOFs with OMS? Also in terms of your results/outcomes?

2) When the authors talk about defective MOFs, are only missing-ligand MOFs considered? Since there are many more types of defects in MOFs, I would suggest the authors be more specific in the main text.

3) The MOFs studied are abbreviated with code names such as POLDUQ. I understand why it is done, but could the authors consider providing (at least in some cases) accepted/common

names along the code names to increase the interest of the experimentalists? Also, a clear statement of how the code names can bring a reader to the structure would be of use too (especially for the people outside the field).

Reviewer: 2

Comments to the Author

The paper describes the generation of a computed dataset of MOFs containing CO_2 and/or H_2O. Several ML models are trained to approximate calculations at the DFT level.

The study has been carefully conducted and the data are available to the public. The paper is carefully written and the topic is extremely relevant. I thus support publication in ACS Central Science once the following aspects have been addressed.

In principle this study represents a step forward compared to generating data with force fields. I don't necessarily disagree with this statement. However, it depends on the force fields used and on the quantum chemistry data quality. There are systematic ways to improve force fields by including parameters derived from first-principle calculations. See for example Ref 44. The authors should comment on these approaches. Perhaps their limitation is that they cannot be used in a high-throughput fashion, but they should still be discussed.

The selection of PBE-D3 as the reference level for DFT calculations is justified, considering the balance between quality and cost. However, it's crucial to explicitly state the associated error with this approximation and clarify the treatment of spin states in open-shell cases. Which spin state did the author study? Additionally, the potential structural changes of adsorbates like CO_2 during the adsorption process and any resulting radical character should be discussed.

In order to study CO_2/H_2O mixtures, the authors performed DFT calculations with at most two water molecules. This is a good start, but one wonders how the framework and the guests will rearrange in the presence of more adsorbate molecules. Moreover, all the DFT calculations presented are static DFT calculations. In other terms no ab initio MD calculations are presented here. I understand that this is very expensive and time consuming, but this aspect should be discussed.

Given the submission to a chemistry journal, it's imperative to highlight the significant findings in terms of chemistry and their potential to inspire new experiments. Mere production of a large dataset should be supplemented with discussions on the chemistry learned and its implications for experimental design.

While active learning offers a promising alternative to traditional force fields, it would be valuable to discuss its computational advantages, including a comparison of the relative costs between force field and neural network methods.

Author's Response to Peer Review Comments:

Dr. David S. Sholl

Director, Transformational Decarbonization Initiative
Oak Ridge National Laboratory, Oak Ridge, TN 37830
Email: shollds@ornl.gov

Professor, School of Chemical & Biomolecular Engineering Georgia Institute of Technology, Atlanta, GA 30332

March 23, 2024

Senior Editor, ACS Central Science

Dear Editor,

My coauthors and I have submitted a revised version of oc-2023-01629m ("The Open DAC 2023 Dataset and Challenges for Sorbent Discovery in Direct Air Capture") for consideration by *ACS Central Science*. The reviewers' comments were very helpful in strengthening and clarifying our manuscript. A reproduction of the reviewers' comments and our point-by-point response is given below. I hope that you will find our revised manuscript is suitable for publication.

Thank you for your consideration,

David Sholl

Transformational Decarbonization Initiative, Oak Ridge National Laboratory

School of Chemical & Biomolecular Engineering, Georgia Institute of Technology

Response to Reviewers for oc-2023-01629m (Sriram et al.)

We appreciate the thoughtful comments from the reviewers. Addressing these comments has strengthened the manuscript. Below we reproduce the reviewers' comments verbatim, along with our point-by-point response.

Reviewer: 1

Recommendation: Publish in ACS Central Science after minor revisions noted.

Sholl and co-workers explore a new ML-driven approach to the discovery of MOF structures interesting for DAC application. They further generate and present a new dataset containing DFT calculations on >8000 MOFs. The amount of work put into this manuscript is very high and the results are very interesting both from the point of view of materials discovery and method optimization. The paper should be considered for publication, however, I have several suggestions from the MOF point of view:

1) The authors talk about the concept of open metal sites (OMS) in MOFs and study their impact on water/CO2 (co-)adsorption. Since contradicting definitions of OMS exist across the literature, it would be important for the authors to define OMS and explain in detail which criteria are used to decide on an MOF having or not having an OMS. As such, the authors also mention that such sites are often "capped" with water, hydroxyl groups or – in reality – solvent molecules left from the synthesis. Does it for example mean that a Ti-O-H moiety is an open metal site?

The brief answer is that we used the definition from the CoRE MOF database. We have modified the text to read "Pristine MOF structures and information regarding the presence of open metal sites in these MOFs are obtained from CoRE MOF database without further modification. Approximately 66% of the pristine MOFs include frameworks with open metal sites."

A longer answer that expands on the specific examples mentioned by the Reviewer is that the OMS detection algorithm from the CoRE MOF work analyzes the first coordination sphere of the metal site. The metal site is classified as an OMS if one of the following criteria are met: 1) the metal is coordinated to three or less atoms; 2) a plane can be found such that the first coordinated atoms

falls either on the plane or on the same side of the plane; 3) a numerical value calculated from bending angles indicates sufficient distortion of the first coordination sphere away from a closed metal site configuration. The OMS classification is solely based on the provided structure file and does not consider experimental conditions. If capped groups or solvent molecules are present near the metal site in the structure file, the site is not an OMS. In this case, Ti-O-H is not considered as an OMS if Ti is also coordinated with more than two atoms from the linker.

Following this, OMS can appear as a result of linker-removal (in defect-containing MOFs) OR can also be a part of the general, undefective structure. Do you think you can provide further insight into the difference between these two classes of MOFs with OMS? Also in terms of your results/outcomes? There is only one category of OMS which inherently is present within the defect-free structure and also persists in the corresponding defective structure. To clarify this point we have modified the text to read "To avoid creating additional OMS following linker removal, metal sites were capped using either a water molecule if the removed linker is charge neutral or hydroxyl(s) if the removed linker was charged to create structures that have no overall charge." We did observe defective MOFs where the metal sites were not properly capped after linker removal. Their occurrence is infrequent, and therefore they are not classified as a distinct subgroup of OMS.

2) When the authors talk about defective MOFs, are only missing-ligand MOFs considered? Since there are many more types of defects in MOFs, I would suggest the authors be more specific in the main text.

We agree that it is a good idea to be more specific on this point. We have modified the text to read "We include two classes of MOF frameworks: *pristine* frameworks and *defective* structures. Although a range of defect types can exist in MOFs, we only considered missing linker defects, since systematic methods exist to add these defects to MOF structures.[85]"

3) The MOFs studied are abbreviated with code names such as POLDUQ. I understand why it is done, but could the authors consider providing (at least in some cases) accepted/common names along the code names to increase the interest of the experimentalists? Also, a clear statement of how the code names can bring a reader to the structure would be of use too (especially for the people outside the field).

We agree that the CSD structure codes, while well defined, are not "user friendly". Only some of the MOFs we identified have common names, but it was a good suggestion to include these names. We have modified the text to read "...and the tables indicate which of the four promising MOF characteristics mentioned above appear in each material. Two of the ten proposed MOFs have common names: PCN-516 for ODIXEG and PCN-46 for LUYHAP. The complete lookup table for common names available for our list of promising MOFs can be found in Table S6."

Additional Questions:

Element of innovation: Top 1%

Impact on the field at large: Top 1%

Strength of authors plan to promote uptake and dissemination of the work: Top 1%

Does the work address an important, unmet need in the chemical community?: Yes

If yes, how important is the unmet need?: Very important

Is this research study suitable for media coverage or a First Reactions (a News & Views piece in the journal)?: Yes

Reviewer: 2

Recommendation: Reconsider after major revisions noted.

The paper describes the generation of a computed dataset of MOFs containing CO2 and/or H2O.

Several ML models are trained to approximate calculations at the DFT level.

The study has been carefully conducted and the data are available to the public. The paper is carefully written and the topic is extremely relevant. I thus support publication in ACS Central Science once the following aspects have been addressed.

We appreciate the reviewer's positive assessment and their thoughtful comments below.

In principle this study represents a step forward compared to generating data with force fields. I don't necessarily disagree with this statement. However, it depends on the force fields used and on the quantum chemistry data quality. There are systematic ways to improve force fields by including parameters derived from first-principle calculations. See for example Ref 44. The authors should comment on these approaches. Perhaps their limitation is that they cannot be used in a highthroughput fashion, but they should still be discussed.

We agree with the reviewer and have modified the discussion of the FF results to clarify this issue. Specifically, we modified the text to read "Given the importance chemisorption in selective CO_2 capture at low concentrations, this finding supports the potential value for ML models for DAC. See Figure S4 for errors in the repulsive region. We note that our FF calculations used "general purpose" FFs that are readily suitable for high throughput calculations. Systematic methods exist to improve classical FFs by including parameters derived from first-principle calculations[44], and the data sets we have introduced may create useful opportunities to develop improved FFs using variations of these methods."

The selection of PBE-D3 as the reference level for DFT calculations is justified, considering the balance between quality and cost. However, it's crucial to explicitly state the associated error with this approximation and clarify the treatment of spin states in open-shell cases. Which spin state did the author

study? Additionally, the potential structural changes of adsorbates like CO2 during the adsorption process and any resulting radical character should be discussed.

We agree with the reviewer that the reference level for DFT should be carefully selected, and spin states matter for open-shell cases. While recognizing the importance of providing the associated error, the extensive number of diverse MOFs we studied coupled with the intricacies of adsorption makes it very difficult to do a comprehensive comparison of different reference levels for DFT calculation. We believe PBE-D3 is a rational choice and have mentioned the following in the text "The generalized gradient approximation (GGA) approach was chosen over more accurate methods such as hybrid functionals or coupled cluster techniques because of the size and diversity of the dataset. Nazarian showed that several different functionals and dispersion corrections perform similarly when making structural and partial charge predictions on a chemically diverse set of MOFs."

To clarify our approach regarding spin states we have modified the text to read "Our calculations included spin polarization to capture spin effects associated with open metal sites, with the simplification that the magnetic moment was initialized as +1 for all atoms. Further exploration of possible spin states may be warranted in cases of special interest." Although we are aware of the possible structural energy differences resulting from different spin states in MOFs with open-shell atoms, there is at least one study showing that spin states have negligible influence on H_2O adsorption energy in CuBTC[Grajciar, L., Bludsky, O., & Nachtigall, P. (2010). Water adsorption on coordinatively unsaturated sites in CuBTC MOF. *The Journal of Physical Chemistry Letters*, *1*(23), 3354-3359]. We are not claiming, however, that this observation will apply to all materials, so the text in the manuscript is intended to simply state how our calculations were performed.

To comment on possible structural changes of the adsorbates we have modified the text to read "It is interesting to ask what attributes give MOFs adsorption energies that are favorable for DAC. We did not conduct an exhaustive analysis of how potential adsorbate structural changes influence adsorption energies due to the relatively rigid nature of CO_2. Instead, we prioritized the examination of MOF motifs which we believe play a more significant role in influencing adsorption energies. Previous research has suggested several characteristics of good candidates …".

In order to study CO2/H2O mixtures, the authors performed DFT calculations with at most two water molecules. This is a good start, but one wonders how the framework and the guests will rearrange in the presence of more adsorbate molecules. Moreover, all the DFT calculations presented are static DFT calculations. In other terms no ab initio MD calculations are presented here. I understand that this is very expensive and time consuming, but this aspect should be discussed.

We agree that the phenomena that can occur with more than two molecules are highly relevant, and had attempted to hint at this in the Impact and Future Outlook section. We have modified this discussion to read "For example, grand canonical Monte Carlo simulations are critical for predicting adsorption isotherms. The models here are untested for this task since they have not seen configurations with more than two adsorbed molecules. It would be very interesting to test predictions of these models against DFT data from higher loadings generated with methods that can sample the full range of possible configurations such as *ab initio* MD."

Although we did not perform ab initio MD calculations (which as noted by the Reviewer tend to be extremely computationally intensive), all of our calculations for coadsorption included full relaxation of the coordinates of adsorbates and MOFs. To emphasize this point we have modified the text to read “In addition to considering the adsorption of single CO_2 and H_2O molecules, we also used DFT to probe the co-adsorption of CO_2 and H_2O in MOFs. Similar to our calculations with single adsorbed molecules, these calculations allowed for full relaxation of the adsorbate and MOF degrees of freedom.”

Given the submission to a chemistry journal, it's imperative to highlight the significant findings in terms of chemistry and their potential to inspire new experiments. Mere production of a large dataset should be supplemented with discussions on the chemistry learned and its implications for experimental design.

We agree that analysis of the relevant chemical motifs is important, and in the original manuscript we primarily addressed this in terms of the presence of four known characteristics that have been previously identified: open-metal sites (OMS), parallel aromatic rings, metal-oxygen-metal (M-O-M) bridging bonds, and under-coordinated nitrogen. We have a brief discussion of these motifs at the bottom of page 9 noting that 93% of MOFs we identified as “promising” have at least one of these. However, the Reviewer’s comment inspired us to take a closer look at which characteristics are most predictive by normalizing their prevalence in promising MOFs by the prevalence in the “general MOF population” (that is, the complete set of MOFs in our dataset). Somewhat surprisingly, this analysis showed that the prevalence of most characteristics in the “promising MOF” population is actually lower than in the “general MOF” population, with the exception of and under-coordinated N and OMSs, which are slightly over-represented in the pristine promising MOF population. This finding is probably not what the Reviewer has in mind to “inspire new experiments”, but we feel that it is a useful way to look at our results to remind readers that it would be easy to over-interpret results and draw simplified mechanistic conclusions that are in fact not entirely supported by the data. We have modified the paragraph in the original text as follows: “224 of the 241 of the promising MOFs can be characterized by at least one of these characteristics. In particular, 72% of the promising pristine MOFs had open metal sites 60% had parallel aromatic rings, and slightly under 30 % had metaloxygen-metal bridges or undercoordinated N atoms. For defective MOFs, open metal sites were slightly less prevalent (54%), while the other motifs occurred with similar frequencies (59% parallel aromatic rings, 34% metal-oxygen-metal bridges, and 26% undercoordinated N atoms). To understand whether these features are truly predictive it is important to compare these results to the prevalence of these features in the total population of pristine/defective MOFs in our data set. In this sense undercoordinated N atoms are the strongest predictor of strong and selective CO_2 adsorption for pristine MOFs, being overrepresented by 30% in the promising MOF population. Open metal sites are also slightly overrepresented (by 10%) in the promising pristine MOF population. However, these characteristics are neither over- or under-represented in defective MOFs. Parallel aromatic rings are underrepresented by 15% in promising pristine and defective MOFs, while metal-oxygen-metal bridges are underrepresented by 21% (pristine) or 31% (defective) MOFs. Many readers will suspect that amine groups can play an important role in creating materials with strong affinity for CO_2. The ODAC23 dataset contains 217 pristine and 254 defective MOFs with an amine functional group. Of these, 7 MOFs (2 pristine and 5 defective) were found to be promising, so amine functional groups were significantly

underrepresented amongst promising MOFs. Details of the frequency normalization calculation can be found in Table S5. These findings suggest while open metal sites and undercoordinated N atoms are the chemical motifs that are most suggestive of strong and selective CO_2 adsorption in pristine MOFs, none of the previously identified motifs are strong indicators for defective MOFs. This suggests that additional development of ideas characterizing the environments enabling strong and selective CO_2 adsorption will be useful in the future."

While active learning offers a promising alternative to traditional force fields, it would be valuable to discuss its computational advantages, including a comparison of the relative costs between force field and neural network methods.

We agree that the comparison of relative costs is relevant, and at the same time note that "cost" comparisons can be made in a number of ways. We have added the following text to the paper, where we endeavor to present information to allow readers to draw their own conclusions:

"**Comparison of computational cost.** Machine learned potentials involve a much higher number of floating point operations than classical force fields. However, most of these operations involve matrix operations which are well suited for modern graphics processing units (GPUs). Therefore, ML models can be run efficiently on a GPU in terms of wall clock time. Since these potentials are often used to run structure relaxations, we computed the average wall clock time per relaxation for UFF, GemNetOC, and EquiformerV2 models across 60 randomly sampled systems from the dataset. We ran the UFF relaxations on a CPU and the ML relaxations on a GPU since those are the most suitable architectures. On average, a UFF relaxation requires 37.67s per CPU core on a Dual Intel Xeon Gold 6226R 2.9 GHz CPU machine. In comparison, GemNet-OC relaxations take 0.8s and EquiformerV2 relaxations take 10.2s on a single 32GB V100 GPU."

Additional Questions:

Element of innovation: High

Impact on the field at large: High

Strength of authors plan to promote uptake and dissemination of the work: High

Does the work address an important, unmet need in the chemical community?: Yes

If yes, how important is the unmet need?: Very important

Is this research study suitable for media coverage or a First Reactions (a News & Views piece in the journal)?: No

Formatting requests from the Editorial Office

TITLE: Please make sure the titles match between the MS title page and the SI title page We have verified that the titles match.

CITATIONS: Please number

All citations in the manuscript are numbered and are referred to by these numbers in the text.

MS FILE: Please unzip, and upload each file separately. We require a Word format for the MS File. Our manuscript was written using LaTeX, not Word. We have provided the LaTeX source for the file(s).

ABSTRACT: Please make sure the word count does not exceed 200 words. We have edited the Abstract to be within this word limit.

TOC MISSING: Provide a TOC image per journal guidelines (3.25 in. × 1.75 in. (8.25 cm × 4.45 cm) ; on the last page of the Manuscript) with the heading "TOC Graphic" above the graphic. Make sure to designate the file as "Graphic for Manuscript." We have made this correction.

SYNOPSIS MISSING: The synopsis should be no more than 200 characters (including spaces) and should reasonably correlate with the TOC graphic. The synopsis is intended to explain the importance of the article to a broader readership across the sciences. Please place your synopsis in the

manuscript file after the TOC graphic, and label it as "Synopsis." The Synopsis has been added following the TOC Graphic.

SI PG#S: The supporting information pages must be numbered consecutively, starting with page S1. We have made this adjustment.

oc-2023-01629m.R2

Name: Peer Review Information for "The Open DAC 2023 Dataset and Challenges for Sorbent Discovery in Direct Air Capture"

Second Round of Reviewer Comments

Reviewer: 2

Comments to the Author

The authors addressed all my comments in a satisfactory way and the paper can now be accepted.

Author's Response to Peer Review Comments:

I have submitted a revised version that has a 60 word Significance Statement replacing the Abstract from the original manuscript. The Synopsis is included at the end of the manuscript below the TOC Graphic.

David
